# Effects of trampoline training on lower-limb strength and balance in children and adolescents with special needs: a multilevel systematic review and meta-analysis

**DOI:** 10.3389/fmed.2026.1808352

**Published:** 2026-05-29

**Authors:** Qingyun Zou, Zhikai Qin, Jianjun Li, Meiqi Xin

**Affiliations:** 1 Provincial University Key Laboratory of Sport and Health Science, School of Physical Education and Sport Science, Fujian Normal University, Fuzhou, Fujian, China; 2 Health Management College of Xianning Polytechnic, Xianning, Hubei, China; 3 School of Physical Education, Hanjiang Normal University, Shiyan, Hubei, China

**Keywords:** adolescents, balance, children with special needs, lower-limb strength, Meta-analysis

## Abstract

**Objective:**

To conduct a thorough assessment of how trampoline training affects lower limb strength and balance in children and adolescents with special needs, while also exploring the relationships between intervention dosage, effect sizes, and potential moderating factors.

**Methods:**

This research adhered to PRISMA standards. A systematic search for randomized controlled trials (RCTs) published through December 10, 2025, was conducted across PubMed, Web of Science, PsycINFO, and the Cochrane Library. Using the PICOS framework, the target population included children and adolescents with special needs; the intervention involved trampoline training; comparisons were made with non-trampoline comparator conditions, including usual care, standard care, physiotherapy, regular physical education, or no intervention; the outcomes measured included lower-limb strength and balance (assessed through standardized scales or objective tests), and the study design involved RCTs. Continuous outcomes were pooled using standardized mean differences (SMDs). A three-level random-effects model was used to account for the dependence of multiple effect sizes within studies. Heterogeneity was assessed using the *Q* test and *I*^2^ statistic. Sensitivity analyses and publication bias assessments, including Egger’s regression and trim-and-fill methods, were carried out. Additionally, subgroup analyses were performed to identify possible moderators.

**Results:**

Fifteen RCTs were analyzed. The combined results indicated that trampoline training significantly enhanced static balance (SMD = 1.47, 95% CI [0.66, 2.28], *p* < 0.001; GRADE: Moderate) and dynamic balance (SMD = 0.72, 95% CI [0.06, 1.37], *p* = 0.032; GRADE: Low). However, the increase in lower-limb strength was not statistically significant (SMD = 0.43, 95% CI [−0.12, 0.97], *p* = 0.125; GRADE: Very Low). All three measures exhibited substantial heterogeneity (*I*^2^ > 75%), and Egger’s tests indicated a potential publication bias. Current evidence most consistently supports trampoline-based training for improving static balance. Evidence for dynamic balance was less stable after bias adjustment, and evidence for lower-limb strength remains insufficient. Subgroup analyses revealed that studies involving longer intervention durations, moderate training frequencies, and primarily children with neurodevelopmental disorders showed greater improvements in balance.

**Conclusion:**

Trampoline training appears to provide the most consistent benefit for static balance in children and adolescents with special needs, whereas evidence for dynamic balance and lower-limb strength remains uncertain. Given the wide variety of populations included, intervention effects may vary across participant types. Further high-quality studies are needed to clarify these effects.

**Systematic review registration:**

https://www.crd.york.ac.uk/PROSPERO/view/CRD420251265788. Unique Identifier: CRD420251265788.

## Introduction

Children and adolescents with special needs are generally defined as those who face enduring challenges in physical, mental, intellectual, or sensory abilities. These challenges can obstruct their learning and social participation because of environmental barriers, requiring additional support. This demographic is important worldwide and has become a central focus for public health and education initiatives (1). It includes various conditions such as autism spectrum disorder (ASD), attention-deficit/hyperactivity disorder (ADHD), cerebral palsy, Down syndrome, and developmental coordination disorder (DCD). The occurrence of ASD is approximately 1 in 160, while ADHD affects about 5 to 7% of school-aged children; DCD also impacts roughly 5% of this group. Cerebral palsy is the most common physical disability in children, with an incidence of around 1 to 4 per 1,000 live births, and Down syndrome occurs in about 1 in 1,000 newborns (2–4). With advancements in diagnostic methods, increased public awareness, and improved survival rates for premature infants and those with congenital conditions, the need for identification and services is growing. In terms of functionality and quality of life, lower-limb strength and balance are vital for basic activities like walking, standing, and jumping, and they are closely linked to independence in daily tasks, fall risk, and long-term musculoskeletal and cardiometabolic health (5). However, children with ASD, cerebral palsy, Down syndrome, and DCD often face motor development challenges and balance or coordination issues, which cause their gross motor and balance skills to be less developed than those of their typically developing peers. This further restricts their ability to participate in daily activities. Therefore, improving lower-limb strength and balance in this group is a key focus in rehabilitation and exercise interventions research (6–9).

Current systematic reviews and meta-analyses show that interventions focused on physical activity can generally improve cardiorespiratory fitness and specific motor skills in children and adolescents with special needs. However, balance is recognized as one of the weakest areas and a key focus for intervention (10). Quantitative studies show that structured exercise programs, which may include strength training (11), balance exercises (12), and multimodal fitness routines (13), can result in notable enhancements in both static and dynamic balance for children with disabilities. Nonetheless, there is significant variability in the types, durations, and intensities of these training programs, making it difficult to establish a clear dose–response relationship (14). Additionally, traditional training methods often depend on repetitive drills or equipment use. Many children with special needs find it hard to sustain attention, motivation, and compliance, which presents practical challenges for engaging them safely.

Trampoline training, which includes both large trampolines and mini-trampoline exercises, has become popular in programs for children and adolescents with special needs. This method merges fun with the advantages of lower-limb engagement. It involves a type of rebound exercise characterized by repeated jumps on a flexible surface. The interaction between the elastic surface and gravity creates a stretch–shortening cycle (SSC) in the lower limb muscles, while also testing balance on an unstable platform. This can improve strength, balance, and heart health (15). Because trampoline surfaces can be variable and unstable, participants must coordinate muscle contractions in their ankles, knees, and hips to keep their posture and process vestibular information, which improves balance and strengthens lower-limb muscles. Research suggests that trampoline training enhances postural control (16–18), motor skills (19–21), and balance (22–24). Nevertheless, the focus on lower-limb strength and balance in this population has often been secondary, with a lack of systematic reviews; the methods for measuring balance vary and are not standardized; and there remains insufficient comprehensive evidence regarding the ideal dosage for trampoline training.

In this study, we assessed lower-limb strength as well as static and dynamic balance as the primary outcomes. Following PRISMA guidelines, we performed a meta-analysis of relevant RCTs. We calculated effect sizes as SMDs using lower-limb strength measurements and standardized outcomes for static and dynamic balance. A three-level random-effects model was employed to aggregate the dependent effect sizes. We assessed heterogeneity using I^2^ and Q tests, while publication bias was examined through sensitivity analyses, Egger’s regression, and the Duval–Tweedie trim-and-fill method. Additionally, subgroup analyses were conducted to compare various intervention formats, such as trampoline training versus standard school activities and trampoline training combined with regular therapy. We also summarized the characteristics of the interventions to provide a comprehensive assessment of the overall effects and identify potentially optimal protocols. Although the included populations consist of diagnostically diverse conditions, they share clinically significant impairments in postural control, balance, coordination, and lower-limb functional performance.

## Materials and methods

### Study design

This study offers a thorough analysis of RCTs and was conducted in accordance with the PRISMA guidelines (25). The study protocol was registered with PROSPERO (Registration No.: CRD420251265788) before selecting studies and followed the principles outlined in the PRISMA statement.

### Study inclusion criteria

The inclusion criteria were as follows: (1) RCTs examining the effect of trampoline training on children and adolescents with special needs; (2) the intervention group must have participated in at least one organized trampoline-based training program, whereas the control group received non-trampoline comparators, such as usual care, physiotherapy, regular physical education, standard care, or no intervention; (3) participants were limited to children and adolescents with special needs, with no restrictions based on gender, ethnicity, or economic background; (4) primary outcomes included standardized assessments of lower-limb strength and balance; and (5) the full text had to be available in English. Excluded studies included non-experimental research (such as theoretical analyses and case studies), non-clinical investigations (including animal or cellular studies), secondary sources (like systematic reviews or meta-analyses), non-original works (such as duplicate articles and conference summaries), non-peer-reviewed gray literature, and interventions that were single-session and lacked structure. Although systematic reviews were not included, their reference lists were examined for potentially relevant studies. Because the review question focused on functional outcomes rather than disease-specific mechanisms, children and adolescents with different special-needs diagnoses were eligible if they met the predefined criteria and reported relevant lower-limb strength or balance outcomes.

### Search strategy

A thorough search was conducted across PubMed, Web of Science, PsycINFO, and the Cochrane Library to identify RCTs evaluating the effects of trampoline training on lower-limb strength and static and dynamic balance in children and adolescents with special needs. The search strategy was organized using the PICOS criteria: population (children and adolescents with special needs), intervention (structured trampoline-based training), comparator (non-trampoline comparator conditions, including usual care, standard care, physiotherapy, regular physical education, traditional therapy, or no intervention), outcomes (improvements in lower-limb strength and balance), and study design (RCTs). Various keywords and subject terms were combined, such as (“trampoline training” OR “rebound therapy” OR “mini-trampoline”) AND (“lower limb strength”) AND (“balance” OR “postural control” OR “static balance” OR “dynamic balance”) AND (RCT OR “Randomized Controlled Trial”). Searches were conducted from inception through December 10, 2025, limited to full-text studies written in English. Additionally, the reference lists of relevant reviews and eligible articles were manually screened to identify any studies that might have been missed. The complete search strategies for all databases are included in the Supplementary material.

### Study selection process

The selection of studies strictly adhered to PRISMA guidelines. All entries were uploaded into Zotero 7.0 to automatically delete duplicates. Two different reviewers screened titles and abstracts to exclude clearly irrelevant studies. Full texts of records that appeared potentially eligible were obtained to verify their eligibility based on PICOS criteria. Any disagreements were resolved through consensus, involving a third reviewer to minimize selection bias. Data extraction was conducted using standardized forms with independent dual entry; key information (such as sample characteristics and intervention details) was verified for accuracy. Any disputed data was resolved through group discussions.

### Data synthesis

All analyses were conducted using R version 4.3.3, with various packages such as meta, metafor, and ggplot2. For outcomes measured continuously, either the mean difference (MD) or the SMD was used, depending on the consistency of the scales; SMDs were adjusted using Hedges’ g (small = 0.2, medium = 0.5, large ≥0.8) (26). When available, the overall mean age of participants was used; if not, the midpoint of the age range was used. To address dependence from multiple effect sizes within a single study, a three-level random-effects model (rma.mv) was used, accounting for variance components related to sampling, within-study, and between-study variances (27). The primary model used restricted maximum likelihood (REML). Heterogeneity was evaluated with the *Q* test (*p* < 0.10 considered significant) and *I*^2^ (>50% indicating substantial heterogeneity) (28). Publication bias was assessed using funnel plots and Egger’s regression, with trim-and-fill methods applied to address asymmetry (29). Influence diagnostics were conducted using standardized residuals (|*Z*| > 2.5) and Cook’s distance (>3 times the mean) to pinpoint influential studies (30). The robustness of the findings was tested through: (1) leave-one-out analysis (metainf); (2) subgroup analyses to investigate sources of heterogeneity. Raw data were transformed into standardized effect sizes using the Hedges–Olkin method $\;\mathrm{SMD}=\frac{M_{\text{Intervention}}-M_{\text{Control}}}{SD_{\text{Pooled}}},\;\;SD_{\text{Pooled}}=\sqrt{\frac{\begin{array}{l} (n_{1}-1)SD_{1}^{2}+\\ (n_{2}-1)SD_{2}^{2} \end{array}}{n_{1}+n_{2}-2}}\;$ (31).

### Outcome definitions and harmonization

Before pooling, outcomes were operationally defined into three specific domains based on construct similarity: lower-limb strength, static balance, and dynamic balance. Lower-limb strength was characterized as outcomes reflecting lower-extremity muscular force, power, or explosive performance (32). Static balance is defined as the ability to maintain postural stability under relatively fixed standing conditions with minimal displacement of the center of mass (33). Dynamic balance is defined as the ability to maintain or restore postural control during movement, locomotion, or tasks involving shifting the center of mass or a changing base of support (34). Outcomes were classified based on construct similarity rather than solely on the specific instrument. Since conceptually similar outcomes were evaluated with different instruments across studies, subgroup analyses by outcome-measure type or scale were preplanned to explore potential heterogeneity caused by differences in assessment tools.

### Risk of Bias (quality) assessment

The Cochrane RoB 2 tool (2019 update) was utilized to evaluate the potential for bias across five areas: (1) the process of randomization; (2) variations from the planned interventions; (3) absence of outcome data; (4) measurement of outcomes; and (5) selection of reported results (35). Two reviewers independently assessed each study, categorizing it as “low risk,” “some concerns,” or “high risk.” Any disagreements were addressed through discussion, and a third reviewer was involved for resolution when needed. Because high risk or concerns about deviations from intended interventions were common across outcomes, these RoB2 findings were subsequently considered in the GRADE assessment, leading to reduced confidence in the pooled estimates—especially for dynamic balance and lower-limb strength.

## Results

### Study selection

**Figure 1:**
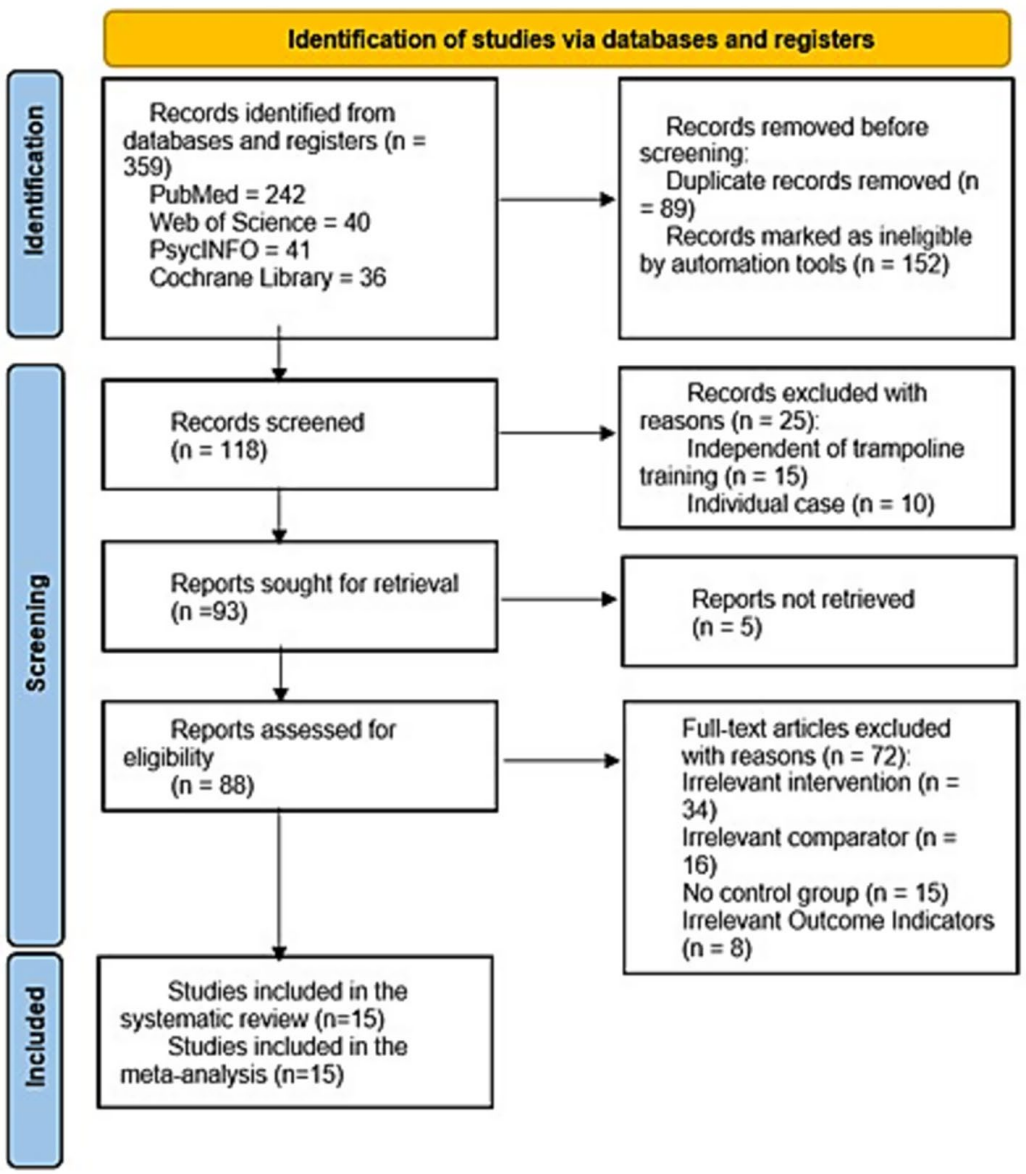
Flow diagram of the selection process.

A total of 359 entries were found across four different databases. After removing 89 duplicates and using automation tools to exclude 152 records, 118 entries remained for title and abstract review (see Figure 1). Of these, 25 were discarded for not meeting the eligibility requirements, leaving 93 reports for retrieval. However, five reports could not be obtained, so 88 full-text articles were evaluated for eligibility. Following the exclusion of 72 full-text articles, 15 RCTs were ultimately included in the final analysis.

### Risk of Bias of included studies

**Figure 2:**
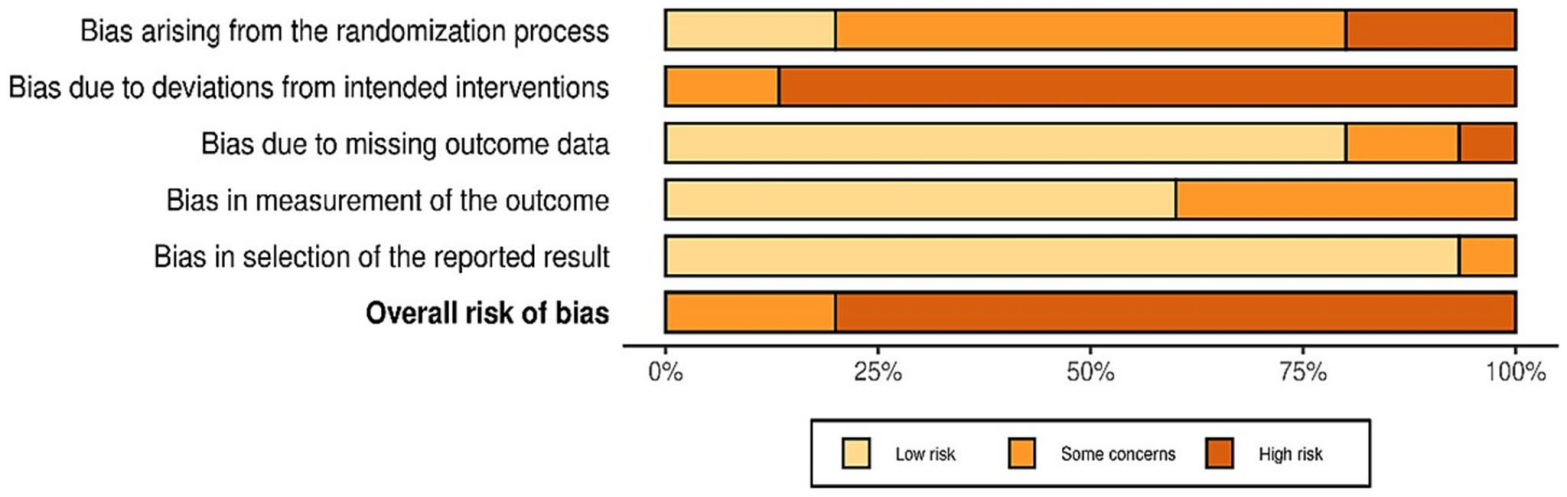
Risk of bias graph.

**Figure 3:**
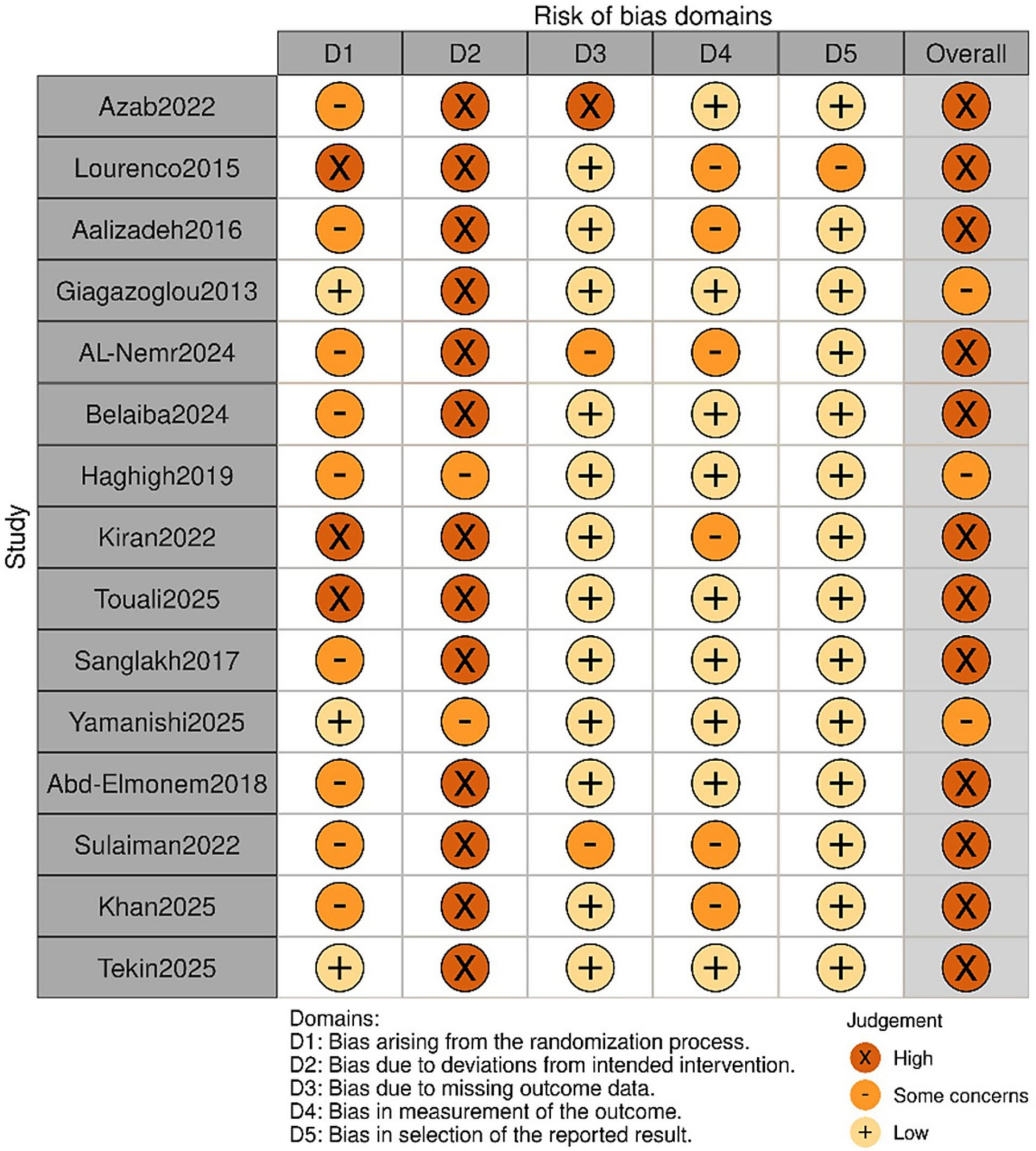
Risk of bias summary.

Most of the included RCTs used random allocation; however, details about sequence generation and allocation concealment were often poorly reported, raising concerns about the validity of the randomization process. Given the characteristics of exercise interventions, achieving blinding was difficult, and deviations from the planned interventions were often observed, leading to a generally higher risk of bias in this area. While the outcome data were mostly complete, indicating a low risk of bias due to missing data overall, a few studies had issues because of poor reporting on how missing data were handled. The risk associated with outcome measurement was generally low, although some studies lacked sufficient details on assessor blinding or measurement consistency. Selective reporting was generally considered low risk, but some studies required careful interpretation because they lacked preregistration or analysis plans. Overall, the high risk in the ‘deviations from intended interventions’ category led to an overall high risk of bias (see Figures 2, 3).

### Study characteristics

A total of fifteen RCTs were analyzed (See Table 1). These studies were carried out in various countries, including Saudi Arabia, Portugal, Iran, Greece, Egypt, Tunisia, Pakistan, Morocco, Japan, and Turkey. The included participants were children and adolescents with diverse neurodevelopmental, intellectual, and neurological conditions, such as Down syndrome (DS), autism spectrum disorder (ASD), intellectual disability (ID), developmental coordination disorder (DCD), and different types of cerebral palsy (e.g., hemiplegic, spastic diplegic, hypotonic; GMFCS levels I–II). The interventions focused on trampoline activities, including stretch-shortening cycle (SSC) training, mini-trampoline workouts, rebound therapy, and multimodal approaches, often combined with physiotherapy (PT), sensory-motor integration, sensory integration therapy, neurodevelopmental therapy, vestibular/proprioceptive and balance training, as well as aerobic exercises. Control groups generally received physiotherapy, standard care, school-based physical education, or no specific intervention. The frequency of sessions usually ranged from 1 to 5 per week over a period of 6 to 32 weeks, with most lasting between 8 and 12 weeks. The outcomes evaluated included lower limb strength and power, balance, and motor skills. Assessment tools used included MFET2, standing long jump (SLJ), Eurofit, EPS pressure platform, handheld dynamometry (HHD), isokinetic devices, Optojump, one-leg stance tests, GMFM-88, MABC-2, BOTMP, Biodex balance system, and PBS.

**Table 1:** Characteristics of the studies in the systematic review and meta-analysis.

Author/year	Country	Design	Sample	Age range	Subject type	Intervention (T/C)	Protocol	Tools
Azab et al., 2022 (40)	Saudi Arabia	RCT	32	7–9 yrs	Children with DS	Trampoline SSC + PT vs. PT	15 min/session, 2×/week, 12 wk	Micro FET2
Lourenço et al., 2015 (43)	Portugal	RCT	16	4–11 yrs	Children with ASD	Mini-trampoline ex. vs. no intervention	45 min/session, 1×/week, 32 wk	SLJ
Aalizadeh et al., 2016 (49)	Iran	RCT	28	11–14 yrs	adolescent students with ID	Trampoline training vs. regular PE	90 min/session, 4×/week, 20 wk	Eurofit
Giagazoglou et al., 2013 (9)	Greece	RCT	18	10.3 ± 1.6 yrs	School-aged children with moderate ID	Trampoline training vs. usual school activities	20 min/session, 5×/week, 12 wk	Eurofit+ EPS
AL-Nemr et al., 2024 (50)	Egypt	RCT	52	5–8 yrs	Children with HCP	Rebound therapy + PT vs. core stability + PT	90 min/session, 3×/week, 12 wk	HHD
Belaiba et al., 2024 (51)	Tunisia	RCT	20	8–11 yrs	Children with mild ASD without ID	Combined fitness training vs. usual activity	60 min/session, 3×/week, 8 wk	IKS + OJS
Haghigh et al., 2019 (52)	Iran	RCT	16	13–17 yrs	Girls with ID	Aerobic ex. + rebound therapy vs. no intervention	30 min/session, 3×/week, 8 wk	Eyes-Closed One-Leg Stance
Kiran Pt et al., 2022 (53)	Pakistan	RCT	22	3–7 yrs.	Children with DS	PNF + PT (mat/ball/trampoline) vs. PT	30 min/session, 3×/week, 12 wk	GMFM-88
Touali et al., 2025 (54)	Morocco	RCT	14	6–12 yrs	Children with ASD (severity level 2/moderate)	SMI program (trampoline/Swiss ball/plyometrics) vs. traditional PE	45 min/session, 3×/week, 15 wk	UQAC-UQAM Battery
Sanglakh et al., 2017 (55)	Iran	RCT	22	7–14 yrs.	Children with LFA	Music-synchronized exercise (including trampoline) vs. same exercise without music	45 min/session, 3×/week, 8 wk	BOTMP
Yamanishi et al., 2025 (56)	Japan	RCT	17	4–8.5 yrs	Children with DCD	ASI vs. usual care	60 min/session, 2×/week, 10 wk	MABC-2
Abd-Elmonem et al., 2018 (57)	Egypt	RCT	40	6–10 yrs	Children with SDCP	PT + balance + rebound exercise vs. PT + balance	60 min/session, 3×/week, 8 wk	Biodex
Sulaiman et al., 2022 (58)	Pakistan	RCT	26	5–12 yrs	Children with DCD	Trampoline jumping + PT vs. PT	Experimental group: 75 min/session; Control group: 60 min/session; 3×/week, 8 wk	PBS
Khan et al., 2025 (59)	Pakistan	RCT	82	5–10 yrs	Children with HTCP	Vestibular stim. (including trampoline) + therapy vs. therapy	30 min/session, 5×/week, 6 wk	PBS
Tekin et al., 2025 (60)	Turkey	RCT	44	2–18 yrs	Children with SCP (GMFCS level I–II)	NDT + PT (including trampoline) vs. PT	45 min/session, 2×/week, 8 wk	PBS

RCT, Randomized Controlled Trial; T/C, Intervention Group/Control Group; FU, Follow-up; wk, weeks; min, minutes; DS, Down syndrome; ASD, autism spectrum disorder; ID, intellectual disability; HCP, hemiplegic cerebral palsy; DCD, developmental coordination disorder; SDCP, spastic diplegic cerebral palsy; LFA, low-functioning autism; HTCP, hypotonic tetraplegic cerebral palsy; SCP, spastic cerebral palsy; SSC, stretch–shortening cycle; PT, physiotherapy; PE, physical education; PNF, Proprioceptive neuromuscular facilitation; SG, Scientific gymnastics; PA, physical activity; SMI, Sensory–motor integration; ASI, Ayres sensory integration; NDT, neurodevelopmental therapy. MFET2, MicroFET 2 Hand-Held Dynamometer; SLJ, Standing Long Jump; Eurofit, Eurofit Physical Fitness Test Battery; EFT, Eurofit Test Battery; EPS, EPS Pressure Platform; HHD, Hand-Held Dynamometer; IKS, Isokinetic Strength System; OJS, Optojump Optical Measurement System; EOSL, Eyes-Closed One-Leg Stance Test; GMFM-88, Gross Motor Function Measure–88; UQAC–UQAM Battery, Université du Québec à Chicoutimi–Université du Québec à Montréal Test Battery; BOTMP, Bruininks–Oseretsky Test of Motor Proficiency; MABC-2, Movement Assessment Battery for Children–Second Edition; BBS, Biodex Balance System; PBS, Pediatric Balance Scale. Outcome domain: lower-limb strength (e.g., MFET2, HHD, IKS, SLJ, Eurofit), static balance (e.g., EOSL, PBS standing items, GMFM-88 standing domain), and dynamic balance (e.g., EPS, Optojump, MABC-2, BOTMP, dynamic Biodex conditions, GMFM-88 locomotion domain).

### Meta-analysis

This meta-analysis examined the effects of trampoline training on lower limb strength and balance in children and adolescents with special needs. The analysis of dynamic balance included 11 studies with 17 effect sizes, while static balance involved 5 studies with 12 effect sizes. Lower-limb strength was assessed in 6 studies with 10 effect sizes, totaling 15 studies overall. Because of variability among the studies (dynamic balance: *I*^2^ = 90.5%, *p* < 0.001; static balance: *I*^2^ = 79.1%, *p* = 0.0075; lower-limb strength: *I*^2^ = 82%, *p* = 0.006), random-effects models were used, with detailed results and visualizations available in the Supplementary file. To address different directions of balance outcomes across various measures (where lower values indicate better performance in some assessments), a direction correction was applied before calculating SMDs to prevent effects from canceling each other out. For outcomes where lower values are preferred, the means were multiplied by −1, while the standard deviations remained unchanged, ensuring consistency in direction. Consequently, positive SMDs were interpreted as improvements in static and dynamic balance resulting from trampoline training.

The combined findings indicated that trampoline training enhanced dynamic balance (SMD = 0.72, 95% CI [0.06, 1.37], *p* = 0.032) and led to a notable improvement in static balance (SMD = 1.47, 95% CI [0.66, 2.28], *p* < 0.001), while showing no significant impact on lower-limb strength (SMD = 0.43, 95% CI [−0.12, 0.97], *p* = 0.125). An evaluation of publication bias and the potential impact of high-impact studies was carried out following the Cochrane Handbook v6.3 (36). Initially, the likelihood of publication bias from small-study effects was assessed using Egger’s regression test. For the assessment of dynamic balance, Egger’s test showed significant publication bias, indicating that the overall estimate might be influenced by heterogeneity and/or small-study effects (*t* = 3.60, *p* = 0.003; see Figure 5A). A significant publication bias was also found for static balance (*t* = 6.54, *p* < 0.001; see Figure 5B). Additionally, for lower-limb strength, Egger’s test indicated a statistically significant publication bias (*t* = 2.54, *p* = 0.035; see Figure 5C).

Subsequently, key studies were identified by combining standardized residuals (|*Z*| > 2.5), Cook’s distance (>3 times the average), and leave-one-out analyses (30). To evaluate the stability of the combined estimates, we performed joint influence diagnostics using Cook’s distance and standardized residuals for static balance, dynamic balance, and lower-limb strength metrics. The findings showed that Sanglakh (2017) for static balance, Belaiba (2024) and AL-Nemr (2024b) for dynamic balance, and AL-Nemr (2024) for lower-limb strength all had Cook’s distances exceeding three times the average threshold, indicating that these studies could significantly influence the combined effects. Nevertheless, the standardized residuals for these studies stayed within ±2.5, so they did not qualify as outliers, indicating their effect sizes were not significantly different from the overall distribution. Following established methodological guidelines for influence diagnostics in meta-analysis, exclusion should only be considered when a study simultaneously meets both high influence criteria (markedly elevated Cook’s distance) and statistical outlier status (|*Z*| exceeding the specified threshold). As a result, we chose not to exclude these studies to avoid selection bias that could result from unnecessary removal of important but credible evidence. To further evaluate the robustness of the pooled effects, we conducted multilevel leave-one-out sensitivity analyses along with combined diagnostics of Cook’s distance and standardized residuals. This analyzed how the pooled effect sizes for static balance, dynamic balance, and lower-limb strength changed after sequentially removing each study. For static balance, the overall pooled effect was SMD = 1.47; after excluding Sanglakh (2017), it adjusted to SMD = 1.11 (see Figure 6A). The effect direction remained consistent, the confidence interval did not cross zero, and the revised estimate stayed within the original 95% CI, indicating that although the study was influential, it did not change the overall conclusion. For dynamic balance, the overall pooled effect was SMD = 0.72; after removing Belaiba (2024), it became SMD = 0.48, and after excluding AL-Nemr (2024b), it was SMD = 0.74 (see Figure 6B). In both cases, the estimates stayed within the 95% CI of the original model and showed only minor variations, indicating that no single study dominated the overall effect. For lower-limb strength, the overall pooled effect was SMD = 0.43; after excluding AL-Nemr (2024), the pooled effect slightly increased to SMD = 0.64. The effect direction remained consistent, and the updated estimate still fell within the original 95% CI, indicating that this study did not artificially inflate the pooled effect; instead, it may have helped stabilize the overall estimate and should not be seen as an outlier requiring exclusion (see Figure 6C).

To assess and address potential publication bias, we performed trim-and-fill analyses on the results for static balance, dynamic balance, and lower-limb strength, which involved estimating missing studies and adjusting the funnel plot’s asymmetry (see Supplementary materials for more details). The findings were as follows: for static balance, the initial pooled effect was SMD = 1.17 (95% CI [0.76, 1.58]). After including three potentially missing studies (k = 15), the bias-corrected estimate decreased to SMD = 0.87 (95% CI [0.34, 1.39], *p* = 0.0012), with *I*^2^ = 67.9%. Compared to the unadjusted model, the effect size decreased by about 0.30 SMD but still remained statistically significant, indicating that trampoline training has a relatively strong positive effect on static balance even after bias adjustments. For dynamic balance, the initial pooled effect was SMD = 0.49 (95% CI [0.07, 1.05]). After imputing five potentially missing studies (k = 22), the adjusted effect size decreased to SMD = −0.02 (95% CI [0.64, 0.61]), *p* = 0.9556. This estimate was substantially weakened and nearly zero, indicating no significant improvement in dynamic balance. This trend aligns with notable small-study effects and/or publication bias, so the unadjusted findings for dynamic balance should be interpreted with caution. Concerning lower-limb strength, the original pooled effect was SMD = 0.51 (95% CI [0.12, 0.91]). After adding three potentially missing studies (*k* = 13), the adjusted estimate dropped to SMD = 0.29 (95% CI [−0.10, 0.68], *p* = 0.1428). The effect size decreased, and the result was no longer statistically significant, further suggesting potential publication bias and/or lack of precision, which requires careful interpretation. In summary, the trim-and-fill adjustment revealed varying levels of susceptibility to bias across outcomes: the effect on static balance stayed relatively consistent, while the pooled estimates for dynamic balance and lower-limb strength were notably more affected by bias. These results highlight the need for larger, well-designed studies with adequate sample sizes and standardized outcome measures to minimize bias and enhance the reliability of evidence in future research.

Additionally, a trial sequential analysis (TSA) was conducted to evaluate the dynamic balance outcomes (see Supplementary materials for more details). The findings showed that the required information size (RIS) for dynamic balance was 42.17, while the accumulated information size was 151.97, resulting in an information ratio of 3.6. This indicates that the current evidence exceeds the predetermined information requirement and, within the TSA framework, provides sufficient data to evaluate the overall impact. The cumulative *Z* value was −4.79, with an absolute value greater than the standard significance threshold (|*Z*| > 1.96), indicating that the evidence for dynamic balance is statistically significant under the sequential monitoring method. We also performed a cumulative meta-analysis based on publication year, including 17 studies (see Supplementary materials). The combined effect size showed a pattern of “initially larger effects followed by a decrease and a trend toward non-significance” as more evidence was added. In the initial phase, the pooled estimate rose steadily and seemed to stabilize around 2022 (k = 6; SMD = 0.93). However, with the addition of later trials—especially those published in 2024 and beyond—the pooled effect size decreased significantly (a drop of 0.409 from the earlier stabilization point). After combining all studies, the overall pooled effect was SMD = 0.52 with a 95% CI [−0.02, 1.06], with the confidence interval crossing zero, indicating that the final estimate was not statistically significant under a random-effects model. The cumulative curve also showed that studies added later were associated with a lower point estimate and a wider (or consistently wide) confidence interval. This suggests that the overall conclusion regarding dynamic balance remains quite uncertain, possibly because of differences among studies, small-study effects, or variations in intervention protocols and outcome measures.

**Figure 4:**
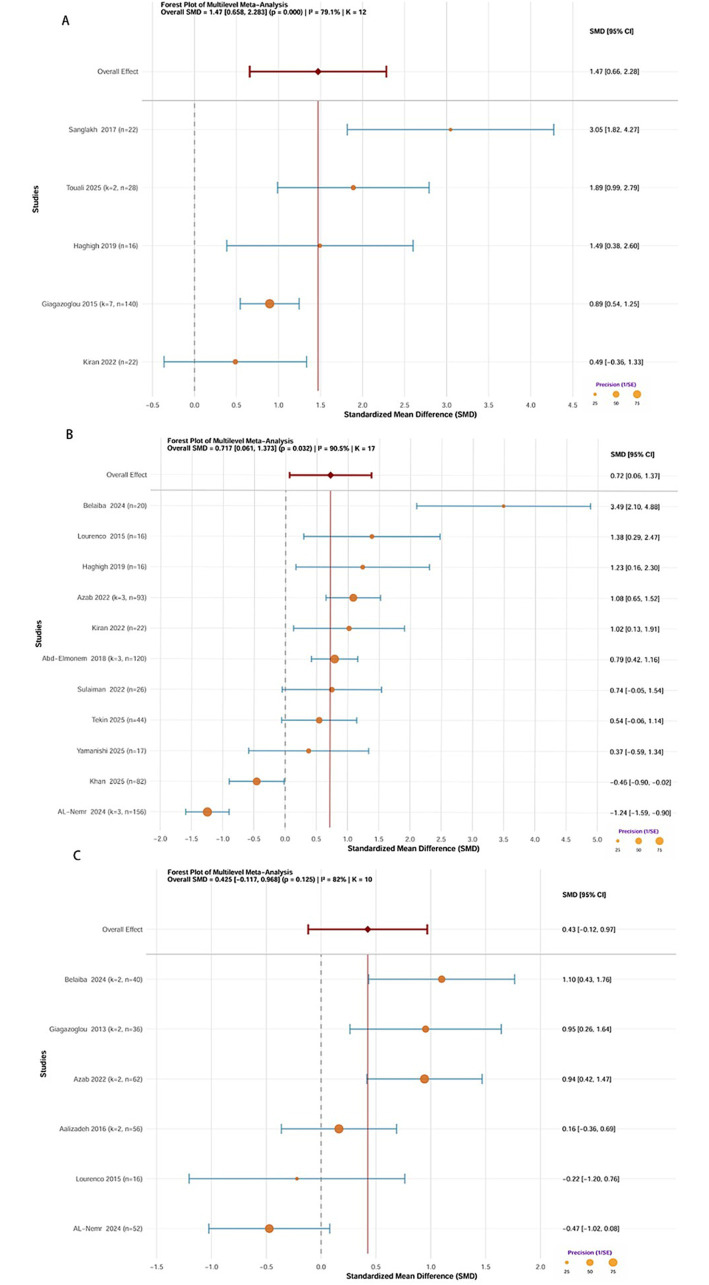
The forest diagram shows the effects of trampoline training on balance and lower limb strength in children and adolescents with special needs. **(A)** Forest plot for dynamic balance, preliminary analysis, including 11 studies (SMD = 0.72, 95% CI: 0.061 to 1.373, *p* = 0.032); **(B)** Forest plot for static balance, preliminary analysis, including 5 studies (SMD = 1.47, 95% CI: 0.658 to 2.283, *p* = 0.000); **(C)** Forest plot for lower-limb strength, preliminary analysis, including 6 studies (SMD = 0.425, 95% CI: −0.117 to 0.968, *p* = 0.125).

Overall, the pooled findings were most robust for static balance, whereas the evidence for dynamic balance and lower-limb strength was more uncertain after bias adjustment. The combined effect on dynamic balance was found to be SMD = 0.72 (95% CI [0.06, 1.37], *p* = 0.032; The corresponding forest plot is presented in Figure 4A), while for static balance, it was SMD = 1.47 (95% CI [0.66, 2.28], *p* < 0.001; The corresponding forest plot is presented in Figure 4B). Conversely, lower-limb strength showed no significant change (SMD = 0.43, 95% CI [−0.12, 0.97], *p* = 0.125; The corresponding forest plot is presented in Figure 4C). Although there was significant variability in the outcomes and potential publication bias was detected (with Egger’s tests suggesting small-study effects), various diagnostic methods—including influence diagnostics using standardized residuals and Cook’s distance, as well as leave-one-out sensitivity analyses—did not identify any individual study that substantially affected the overall results. This suggests that, despite some uncertainty, the overall direction of the pooled effects remains fairly consistent. An integrated assessment using the GRADE framework provides moderate-certainty evidence for a significant clinical benefit of trampoline training in improving static balance for this group. In contrast, the current evidence regarding dynamic balance and lower-limb strength remains uncertain and does not convincingly support a clear benefit of trampoline training in these areas. This difference in certainty was mainly due to the RoB 2 findings, which revealed that many included trials had significant methodological limitations, especially a high risk of bias from deviations from intended interventions (D2), along with some concerns in the randomization process (D1) and, in several studies, outcome measurement (D4). Additionally, substantial residual heterogeneity further lowered confidence in the pooled estimates, especially for dynamic balance and lower-limb strength. Detailed outcome-specific GRADE judgments and downgrade rationales are provided in the Supplementary material.

**Figure 5:**
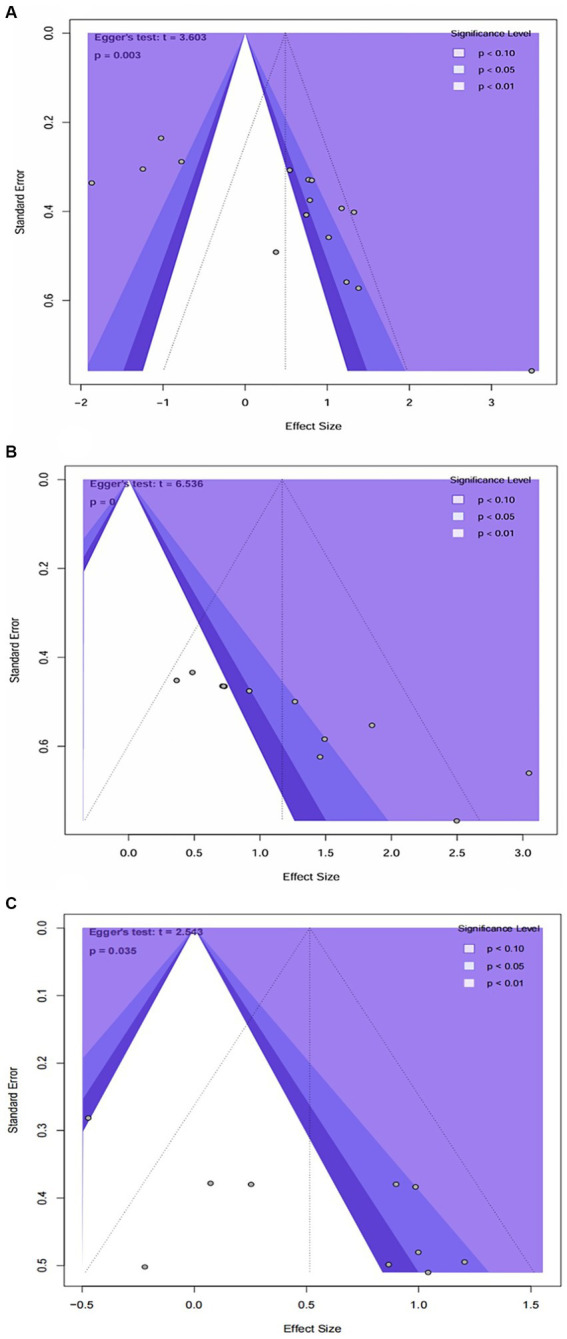
Funnel plots combined with Egger regression tests have published bias in studies evaluating the effects of trampoline training on balance and lower extremity strength. **(A)** Dynamic balance (*t* = 3.603, *p* = 0.003). **(B)** Static balance (*t* = 6.536, *p* < 0.001). **(C)** Lower-limb strength (*t* = 2.543, *p* = 0.035).

**Figure 6:**
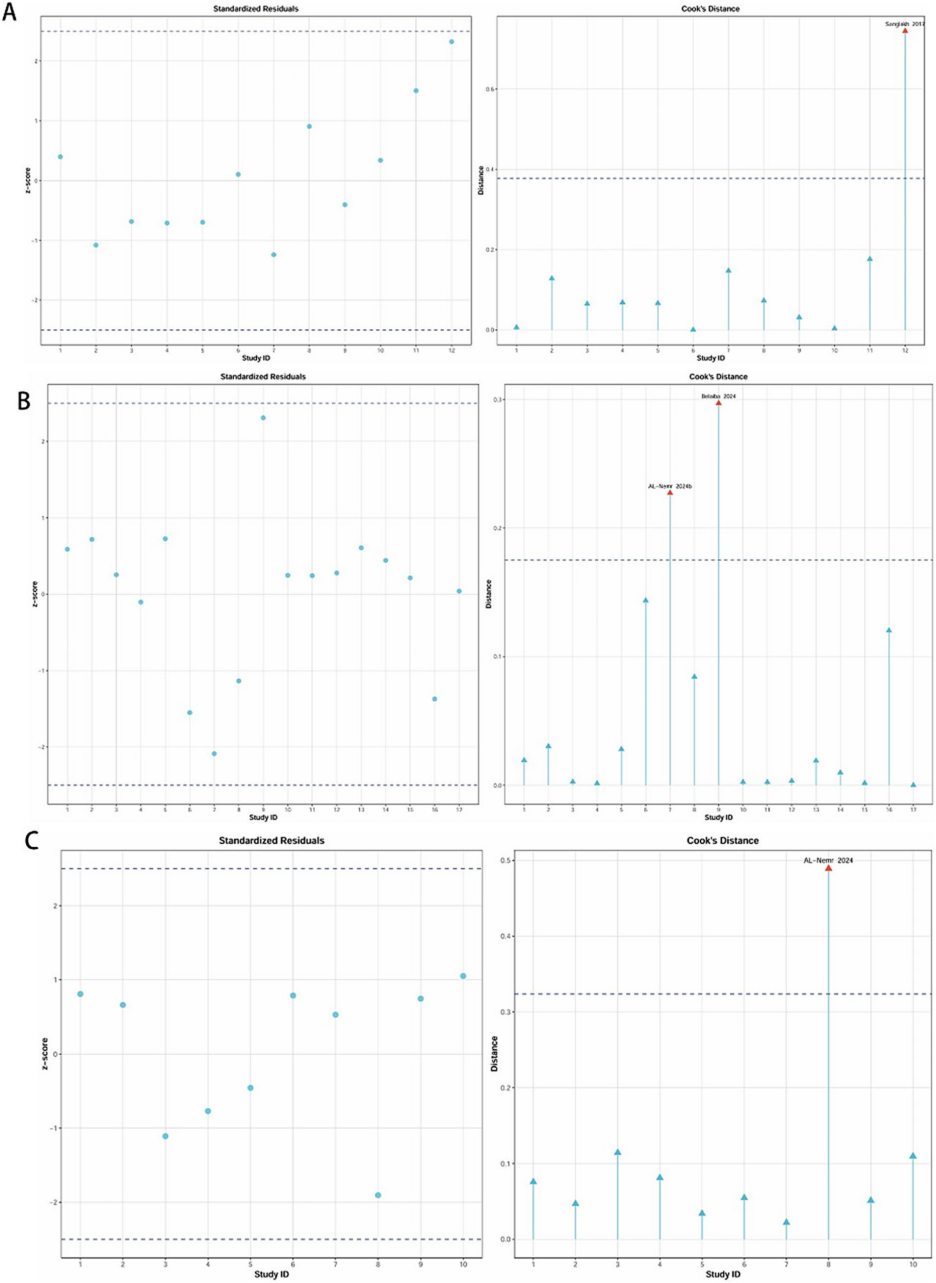
Influence diagnostics for included studies using standardized residuals and Cook’s distance. **(A)** Influence diagnostics for static balance. **(B)** Influence diagnostics for dynamic balance. **(C)** Influence diagnostics for lower-limb strength. In each panel, the left plot shows standardized residuals versus study ID, and the right plot shows Cook’s distance versus study ID.

### Subgroup analysis

Subgroup analyses were predefined and performed to examine potential moderators of the effects of trampoline training on both static and dynamic balance (see Table 2). The selected moderator variables included: (1) age group; (2) participant type; (3) intervention format; (4) training frequency; (5) intervention duration; (6) session length; (7) methodological quality, assessed using the Cochrane Risk of Bias tool (RoB 2); and (8) outcome-measure type/scale type. Because similar outcomes were measured with different instruments across studies, subgroup analyses by outcome-measure type were planned in advance to check if measurement differences might affect the overall estimates. The certainty of evidence for each subgroup estimate was evaluated using the GRADE (Grading of Recommendations Assessment, Development and Evaluation) (37) framework. Because several subgroup estimates were based on very small numbers of studies (*k* ≤ 2), these subgroup analyses should be considered exploratory and interpreted with caution.

**Table 2:** Subgroup analysis of trampoline training at the level of static and dynamic balance in children and adolescents with special needs.

Scale type	Dimensionality	K	*N*	*I*^2^ (%)	Effect model	SMD/MD (95% CI)	GRADE	*p*
Static balance	Motion frequency/week							
	3	12	228	54	Random	1.24(0.81,1.68)	Moderate	0.000
	Intervention cycle/week							
	8 weeks	2	38	69.4	Random	2.34(0.79,3.90)	Low	0.003
	12 weeks	8	162	0.0%	Random	0.86(0.54,1.19)	Moderate	0.000
	15 weeks	2	28	21.4	Random	2.03(0.95,3.11)	Low	0.000
	Intervention time/min							
	45 min	10	190	57.3	Random	1.31(0.81,1.81)	Moderate	0.000
	30 min	2	38	54.3	Random	0.97(−0.07,2.02)	Very low	0.068
	Subject type							
	ID	1	16	*	Random	1.58(0.44,2.72)	Low	0.007
	DS	1	22	*	Random	0.51(−0.05,0.61)	Very low	0.244
	DCD	7	140	0.0%	Random	0.93(0.57,1.28)	Moderate	0.000
	ASD	3	50	39.7	Random	2.43(1.44,3.42)	Moderate	0.000
Dynamic balance	Intervention mode							
	Trampoline+ Other vs. Control	12	228	54.0	Random	1.24(0.81,1.68)	Moderate	0.000
	Object age							
	Adolescents	1	16	*	Random	1.58(0.44,2.72)	Low	0.007
	Children	11	212	57.0	Random	1.22(0.75,1.69)	Moderate	0.000
	Scale type							
	Functional task / Scale-based	5	88	71.4	Random	1.81(0.83,2.79)	Low	0.000
	Instrument-based	7	140	0.0%	Random	0.93(0.57,1.28)	Moderate	0.000
	Literature quality assessment							
	Some concerns	1	16	*	Random	1.58(0.44,2.72)	Low	0.007
	High concerns	11	212	57.0	Random	1.22(0.75,1.69)	Moderate	0.000
	Motion frequency/week							
	2	5	154	5.8	Random	0.86(0.51,1.20)	Moderate	0.000
	1	1	16	*	Random	1.46(0.34,2.58)	Low	0.011
	3	10	360	92.0	Random	0.44(−0.38,1.25)	Very low	0.294
	5	1	82	*	Random	−0.46(−0.9, −0.02)	Low	0.039
	Intervention cycle/week							
	12 weeks	7	271	92.9	Random	0.05(−0.94,1.04)	Very low	0.926
	10 weeks	1	17	*	Random	0.40(−0.57,1.36)	Very low	0.422
	32 weeks	1	16	*	Random	1.46(0.34,2.58)	Low	0.011
	8 weeks	7	226	61.0	Random	1.01(0.54,1.48)	Moderate	0.000
	6 weeks	1	82	*	Random	−0.46(−0.9, −0.02)	Low	0.039
	Intervention time/min							
	15 min	3	93	0.0%	Random	1.11(0.67,1.55)	Moderate	0.000
	60 min	6	183	65.9	Random	1.00(0.44,1.56)	Moderate	0.000
	45 min	2	60	49.0	Random	0.88(0.03,1.73)	Moderate	0.044
	90 min	3	156	68.2	Random	−1.3(−1.92, −0.68)	Low	0.000
	30 min	3	120	87.6	Random	0.57(−0.69,1.82)	Very low	0.376
	Subject type							
	ID	1	16	*	Random	1.31 (0.21, 2.40)	Low	0.019
	DS	4	115	0.0%	Random	1.10 (0.71, 1.50)	Moderate	0.000
	DCD	2	43	0.0%	Random	0.62(0.00, 1.23)	Very low	0.050
	ASD	2	36	81.2	Random	2.50 (0.36, 4.63)	Low	0.022
	HCP	3	156	68.2	Random	−1.3 (−1.92, −0.68)	Very low	0.000
	SDCP	3	120	0.0%	Random	0.80 (0.43, 1.18)	Moderate	0.000
	SCP	1	44	*	Random	0.55(−0.05, 1.16)	Very low	0.073
	HTCP	1	82	*	Random	−0.46 (−0.9, −0.02)	Very low	0.039
	Intervention mode							
	Trampoline+ Other vs. Control	16	596	89.4	Random	0.49 (−0.05, 1.03)	Very low	0.075
	Trampoline vs. Control	1	16	*	Random	1.46 (0.34, 2.58)	Low	0.011
	Object age							
	Adolescents	2	60	28.5	Random	0.79 (0.10, 1.47)	Very low	0.092
	Children	15	552	90.1	Random	0.50 (−0.08, 1.09)	Low	0.024
	Scale type							
	Functional task/ Scale-based	8	243	83.5	Random	0.96 (0.24, 1.67)	Very low	0.009
	Instrument-based	9	369	92.0	Random	0.19 (−0.59, 0.97)	Very low	0.632
	Literature quality assessment							
	Some concerns	14	535	90.8	Random	0.51 (−0.12, 1.13)	Very low	0.104
	High concerns	3	77	0.0%	Random	0.65 (0.19, 1.11)	Moderate	0.006

K, number of studies; N, total sample size; *I*^2^, *I*-squared statistic (heterogeneity); SMD, standardized mean difference; CI, confidence interval; DS, Down syndrome; ASD, autism spectrum disorder; ID, intellectual disability; DCD, developmental coordination disorder; HCP, hemiplegic cerebral palsy; SDCP, spastic diplegic cerebral palsy; SCP, spastic cerebral palsy; HTCP, hypotonic tetraplegic cerebral palsy; min, minutes; wk, weeks; *Not applicable (N/A).

Most subgroups showed notable positive effects on static balance. Significant improvements were observed for 3 sessions per week (SMD = 1.24, *p* < 0.001, GRADE: Moderate), intervention durations of 8 weeks (SMD = 2.34, *p* = 0.003, GRADE: Low), 12 weeks (SMD = 0.86, *p* < 0.001, GRADE: Moderate), and 15 weeks (SMD = 2.03, *p* < 0.001, GRADE: Low), and 45 min per session (SMD = 1.31, *p* < 0.001, GRADE: Moderate), while 30 min per session was not significant (SMD = 0.97, *p* = 0.068, GRADE: Very Low). By participant type, significant effects were observed in ID (SMD = 1.58, *p* = 0.007, GRADE: Low), DCD (SMD = 0.93, *p* < 0.001, GRADE: Moderate), and ASD (SMD = 2.43, *p* < 0.001, GRADE: Moderate), but not in DS (SMD = 0.51, *p* = 0.244, GRADE: Very Low). Significant effects were also observed for trampoline plus other interventions versus control (SMD = 1.24, *p* < 0.001, GRADE: Moderate), in both adolescents (SMD = 1.58, *p* = 0.007, GRADE: Low) and children (SMD = 1.22, *p* < 0.001, GRADE: Moderate), in functional task/scale-based measures (SMD = 1.81, *p* < 0.001, GRADE: Low) and instrument-based measures (SMD = 0.93, *p* < 0.001, GRADE: Moderate), and in studies rated as having some concerns (SMD = 1.58, *p* = 0.007, GRADE: Low) or high concerns (SMD = 1.22, *p* < 0.001, GRADE: Moderate).

For dynamic balance, the subgroup pattern was more diverse. Significant positive effects were seen for 2 sessions/week (SMD = 0.86, *p* < 0.001, GRADE: Moderate) and 1 session/week (SMD = 1.46, *p* = 0.011, GRADE: Low), while 3 sessions/week was not significant (SMD = 0.44, *p* = 0.294, GRADE: Very Low). Conversely, 5 sessions/week showed a significant negative effect (SMD = −0.46, *p* = 0.039, GRADE: Low). Significant benefits were observed for intervention durations of 8 weeks (SMD = 1.01, *p* < 0.001, GRADE: Moderate) and 32 weeks (SMD = 1.46, *p* = 0.011, GRADE: Low), whereas 10 weeks (SMD = 0.40, *p* = 0.422, GRADE: Very Low) and 12 weeks (SMD = 0.05, *p* = 0.926, GRADE: Very Low) showed no significant effects; 6 weeks showed a significant negative effect (SMD = −0.46, *p* = 0.039, GRADE: Low). In terms of session length, significant positive effects were observed for 15 min/session (SMD = 1.11, *p* < 0.001, GRADE: Moderate), 60 min/session (SMD = 1.00, *p* < 0.001, GRADE: Moderate), and 45 min/session (SMD = 0.88, *p* = 0.044, GRADE: Moderate). In contrast, 30 min/session was not significant (SMD = 0.57, *p* = 0.376, GRADE: Very Low), and 90 min/session showed a significant negative effect (SMD = −1.30, *p* < 0.001, GRADE: Low). By participant type, significant positive effects were observed in ID (SMD = 1.31, *p* = 0.019, GRADE: Low), DS (SMD = 1.10, *p* < 0.001, GRADE: Moderate), ASD (SMD = 2.50, *p* = 0.022, GRADE: Low), and SDCP (SMD = 0.80, *p* < 0.001, GRADE: Moderate). DCD was borderline significant (SMD = 0.62, *p* = 0.050, GRADE: Very Low), and SCP was not significant (SMD = 0.55, *p* = 0.073, GRADE: Very Low), while HCP (SMD = −1.30, *p* < 0.001, GRADE: Very Low) and HTCP (SMD = −0.46, *p* = 0.039, GRADE: Very Low) showed significant negative effects. Additionally, trampoline versus control was significant (SMD = 1.46, *p* = 0.011, GRADE: Low), while trampoline plus other interventions versus control was not (SMD = 0.49, *p* = 0.075, GRADE: Very Low). Significant effects were also observed for functional task/scale-based measures (SMD = 0.96, *p* = 0.009, GRADE: Very Low) and studies with high concerns (SMD = 0.65, *p* = 0.006, GRADE: Moderate), whereas instrument-based measures (SMD = 0.19, *p* = 0.632, GRADE: Very Low) and studies rated as some concerns (SMD = 0.51, *p* = 0.104, GRADE: Very Low) were not significant. Overall, the subgroup results were more consistent for static balance than for dynamic balance.

## Discussion

The current meta-analysis revealed that trampoline training significantly enhanced static balance in children and adolescents with special needs (SMD = 1.47, *p* < 0.001, GRADE: Moderate) and also demonstrated a beneficial effect on dynamic balance in the primary model (SMD = 0.72, *p* = 0.032, GRADE: Low), while no significant improvement was seen for lower-limb strength (SMD = 0.43, *p* = 0.125, GRADE: Very Low). However, after trim-and-fill adjustment, the effect for static balance remained significant (SMD = 0.87, *p* = 0.0012, GRADE: Moderate), while the effect for dynamic balance was greatly reduced and became not significant (SMD = −0.02, *p* = 0.9556, GRADE: Low); lower-limb strength also remained non-significant (SMD = 0.29, *p* = 0.1428, GRADE: Very Low). Taken together, these findings suggest that the most consistent benefit of trampoline training in this population is improved postural stability rather than force-related outcomes or more challenging dynamic motor tasks (38). This pattern is biomechanically plausible because repeated jumping and landing on an unstable elastic surface constantly challenge the stretch–shortening cycle, inter-joint coordination, vestibular integration, and reactive postural control (22, 39).

These findings should also be considered within the broader context of exercise interventions for children with disabilities. Previous systematic reviews and meta-analyses have demonstrated that exercise interventions can enhance balance and related motor performance in children and adolescents with disabilities, particularly those with intellectual disabilities and other neurodevelopmental disorders (10, 14). Additionally, strength training, balance training, and multimodal exercise programs have been shown to enhance static and dynamic balance in children with motor impairments (11, 12). Within this broader rehabilitation literature, the present study indicates that trampoline training can be considered a feasible exercise modality, but its effects seem to be more consistent for static balance than for dynamic balance or lower-limb strength.

The subgroup analyses further support this interpretation. For static balance, clearer positive effects were observed in DCD (SMD = 0.93, *p* < 0.001, GRADE: Moderate) and ASD (SMD = 2.43, *p* < 0.001, GRADE: Moderate), and a significant effect was also found in ID (SMD = 1.58, *p* = 0.007, GRADE: Low), although this estimate was based on only one study. These findings are broadly consistent with previous studies showing that trampoline training or similar elastic-support-surface interventions can improve balance and motor performance in children with Down syndrome, intellectual disability, and developmental coordination disorder (40, 41), as well as with evidence that motor-based interventions improve balance and motor performance in children with DCD (42). Exercise-related benefits for balance have also been reported in children with ASD (43, 44). By contrast, the DS subgroup was not significant for static balance (SMD = 0.51, *p* = 0.244, GRADE: Very Low), indicating that the evidence for this specific outcome remains limited despite encouraging findings from broader exercise-based reviews in children with Down syndrome (45). For dynamic balance, the subgroup pattern was more heterogeneous. Significant positive effects were observed in DS (SMD = 1.10, *p* < 0.001, GRADE: Moderate), ASD (SMD = 2.50, *p* = 0.022, GRADE: Low), SDCP (SMD = 0.80, *p* < 0.001, GRADE: Moderate), and ID (SMD = 1.31, *p* = 0.019, GRADE: Low). By contrast, DCD showed only borderline significance (SMD = 0.62, *p* = 0.050, GRADE: Very Low), SCP was not significant (SMD = 0.55, *p* = 0.073, GRADE: Very Low), and negative subgroup estimates were observed in HCP (SMD = −1.30, *p* < 0.001, GRADE: Very Low) and HTCP (SMD = −0.46, *p* = 0.039, GRADE: Very Low). This pattern indicates that dynamic balance is more affected than static balance by differences in disability subtype, baseline motor function, and task-specific demands. The positive findings in DS are in line with recent evidence that physical exercise can improve dynamic and overall balance in children with Down syndrome (45), while the favorable signals in ASD are consistent with meta-analytic evidence supporting exercise-related improvements in balance in this group (44). The mixed results across cerebral palsy-related subgroups suggest that the response to trampoline training in CP varies; this may be due to differences in subtype, severity, gait limitations, muscle tone, and concurrent rehabilitation. Previous syntheses have also indicated that exercise interventions can enhance balance in children with CP overall, but the extent of benefit differs across modalities and participant characteristics (46). Intervention dosage and measurement approach may also explain part of the observed heterogeneity. For static balance, the effects were more stable under moderate protocols, especially 3 sessions per week (SMD = 1.24, *p* < 0.001, GRADE: Moderate), 12 weeks (SMD = 0.86, *p* < 0.001, GRADE: Moderate), and 45 min per session (SMD = 1.31, *p* < 0.001, GRADE: Moderate). Positive effects on dynamic balance were observed at 2 sessions per week (SMD = 0.86, *p* < 0.001, GRADE: Moderate), after 8 weeks (SMD = 1.01, *p* < 0.001, GRADE: Moderate), and with session durations of 15 min (SMD = 1.11, *p* < 0.001, GRADE: Moderate), 45 min (SMD = 0.88, *p* = 0.044, GRADE: Moderate), and 60 min (SMD = 1.00, *p* < 0.001, GRADE: Moderate). In contrast, unfavorable signals were observed in 90 min/session (SMD = −1.30, *p* < 0.001, GRADE: Low) and 5 sessions/week (SMD = −0.46, *p* = 0.039, GRADE: Low), which may suggest that excessive training load can cause fatigue, decrease movement quality, and impair postural regulation in children with limited motor reserve (47, 48). Furthermore, in the dynamic-balance analysis, functional task/scale-based measures showed a significant effect (SMD = 0.96, *p* = 0.009, GRADE: Very Low), while instrument-based measures did not (SMD = 0.19, *p* = 0.632, GRADE: Very Low), indicating that different tools may assess different aspects of balance performance and vary in sensitivity to change. Besides these clinical and methodological differences, the significant heterogeneity probably also stemmed from variability in combined intervention formats, small-study effects, and the overall increased risk of bias due to deviations from planned interventions.

Overall, the available evidence supports trampoline training as an effective balance-focused intervention for children and adolescents with special needs, especially for static balance. However, since several subgroup estimates were derived from very few studies and many were rated as low or very low certainty, these subgroup findings should be considered exploratory rather than conclusive. Future randomized controlled trials should incorporate clearer phenotypic stratification, larger sample sizes, more standardized training protocols, and more consistent balance measures to identify which populations benefit most and under what conditions.

## Conclusion

This multilevel systematic review and meta-analysis suggests that trampoline training may offer relatively consistent benefits for static balance in children and adolescents with special needs (SMD = 1.47, *p* < 0.001, GRADE: Moderate), and this effect remained significant after bias adjustment (SMD = 0.87, *p* = 0.0012). In contrast, although the primary analysis indicated a possible benefit for dynamic balance (SMD = 0.72, *p* = 0.032, GRADE: Low), this effect was not confirmed after trim-and-fill adjustment. No clear beneficial effect was found for lower-limb strength in either the primary analysis or the bias-adjusted model. Subgroup analyses further showed that the response to trampoline training differed across participant groups and intervention settings. Clearer positive trends were seen in DCD and ASD for static balance, and in DS, ASD, and SDCP for dynamic balance, while results across cerebral palsy subtypes were less consistent. These results should be interpreted carefully because the included studies varied in participant characteristics, intervention dosage, combined therapies, and outcome measures. Overall, trampoline training seems to have practical value as a balance-focused intervention, especially for postural stability, but its optimal dosage and population-specific usefulness remain uncertain. More high-quality, adequately powered, and better standardized trials are necessary to improve future clinical guidelines.

## Limitations

This study has several limitations. First, while including multiple special populations enabled a broader assessment of the potential applicability of trampoline training, it also introduced significant clinical heterogeneity. Differences in pathological features, functional limitations, and rehabilitation goals among various disability types may cause varied responses to similar intervention protocols, which likely explains inconsistent some findings. Second, the total number of included studies was limited, and several subgroup analyses were based on small samples, which decreased the stability of the conclusions, especially for dynamic balance and lower limb strength. Third, significant variation across studies in training frequency, intervention duration, session length, combined intervention formats, and outcome measurement tools made it harder to interpret the pooled results. Additionally, some outcomes showed signs of publication bias and small study effects, and the generally high risk of bias, especially bias related to deviations from intended interventions, may have further decreased confidence in the pooled estimates, particularly for dynamic balance and lower limb strength. Future studies should implement more stratified designs, target specific disability subtypes, and use larger samples, more standardized intervention protocols, and more consistent outcome measures so that the findings can better support generalizable and individualized recommendations.

## Data Availability

The original contributions presented in the study are included in the article/Supplementary material, further inquiries can be directed to the corresponding author/s.
