# Supplementary material for: Effects of trampoline training on lower-limb strength and balance in children and adolescents with special needs: a multilevel systematic review and meta-analysis
Source: Front Med (Lausanne). 2026 May 29;13:1808352. doi: 10.3389/fmed.2026.1808352 (PMC13259865; doi:10.3389/fmed.2026.1808352)
Supplement: Supplementary file 1 [file Data_Sheet_1.DOCX]

Effects of Trampoline Training on Lower-Limb Strength and Balance in Children and Adolescents with Special Needs: A Multilevel Systematic Review and Meta-analysis

Supplementary Material

[**Supplementary document Retrieval Strategy** 2](#_Toc224929713)

[**Quality Assessment** 4](#_Toc224929714)

[**Static balance** 5](#_Toc224929715)

[Egger’s Test Funnel Plot for Publication Bias Assessment. 5](#_Toc224929716)

[Influence diagnostics for included studies using standardized residuals and Cook’s distance. 5](#_Toc224929717)

[Figure of the Initial Sensitivity Analysis. 6](#_Toc224929718)

[Trim-and-Fill Funnel Plot 6](#_Toc224929719)

[Summary of Findings — The effect of trampoline training on static balance ability in children and adolescents with special needs. 7](#_Toc224929720)

[**Dynamic balance** 9](#_Toc224929721)

[Egger’s Test Funnel Plot for Publication Bias Assessment. 9](#_Toc224929722)

[Influence diagnostics for included studies using standardized residuals and Cook’s distance. 9](#_Toc224929723)

[Figure of the Initial Sensitivity Analysis. 10](#_Toc224929724)

[Trim-and-Fill Funnel Plot 10](#_Toc224929725)

[Summary of Findings — Effects of trampoline training on dynamic balance ability of children and adolescents with special needs 10](#_Toc224929726)

[Dynamic equilibrium sequential test analysis and cumulative meta-analysis diagram. 12](#_Toc224929727)

[**Lower limb strength** 13](#_Toc224929728)

[Egger’s Test Funnel Plot for Publication Bias Assessment. 13](#_Toc224929729)

[Influence diagnostics for included studies using standardized residuals and Cook’s distance. 13](#_Toc224929730)

[Figure of the Initial Sensitivity Analysis. 14](#_Toc224929731)

[Trim-and-Fill Funnel Plot 14](#_Toc224929732)

[Summary of Findings — Effects of trampoline training on lower limb strength ability in children and adolescents with special needs 15](#_Toc224929733)

**Supplementary document Retrieval Strategy**

| **PubMed** | ("Trampolining"[Mesh] OR "Exercise Therapy"[Mesh]  OR trampoline*[tiab] OR "trampoline training"[tiab] OR "trampoline exercise*"[tiab]  OR "mini-trampoline"[tiab] OR "rebound exercise"[tiab] OR "rebound training"[tiab]  OR "rebound therapy"[tiab])  AND  ("Motor Activity"[Mesh] OR "Physical Fitness"[Mesh] OR "Exercise Test"[Mesh]  OR "Muscle Strength"[Mesh] OR "Postural Balance"[Mesh] OR "Gait"[Mesh]  OR "motor performance"[tiab] OR "motor function"[tiab] OR "motor ability"[tiab]  OR "physical performance"[tiab] OR "functional performance"[tiab]  OR "exercise capacity"[tiab] OR "exercise performance"[tiab]  OR balance[tiab] OR "postural control"[tiab]  OR strength[tiab] OR "muscle strength"[tiab] OR "muscle power"[tiab])  AND  ("Randomized Controlled Trial"[pt] OR "Controlled Clinical Trial"[pt] OR "Clinical Trial"[pt]  OR trial[tiab] OR random*[tiab] OR "control group"[tiab]  OR "controlled study"[tiab] OR "intervention study"[tiab] OR intervention*[tiab])  NOT  ("Review"[pt] OR "Case Reports"[pt] OR "Comment"[pt])Filters: |
| --- | --- |
| **Web of Science** | (TS=("trampolin*" OR "trampoline training" OR "trampoline exercise*" OR "mini-trampoline" OR "rebound exercise" OR "rebound training" OR "rebound therapy")  AND  TS=("motor ability" OR "motor performance" OR "motor function" OR "physical fitness" OR "physical performance" OR "functional performance" OR "exercise capacity" OR "balance" OR "postural balance" OR "postural control" OR "muscle strength" OR "muscle power" OR "leg strength" OR "lower limb" OR gait)  AND  TS=("randomized controlled trial" OR RCT OR "controlled trial" OR "controlled study" OR "intervention study" OR intervention* OR "training program" OR "exercise program")  NOT  TS=("review" OR "case report" OR "systematic review" OR "meta-analysis") ) |
| **PsycINFO** | exp Trampolining/ OR trampoline OR "trampoline training" OR "trampoline exercise OR "mini-trampoline".ti,ab.AND exp Muscle Strength/OR exp Postural Balance/ OR exp Physical Fitness/OR ("muscle strength" OR "muscle power" OR "balance" OR "postural control"OR "physical fitness" OR "physical performance").ti,ab. AND exp Randomized Controlled Trials/ OR Controlled Clinical Trials as Topic/OR RCT. OR random OR "controlled trial"OR "control group".ti,ab. |
| **Cochrane Library** | [mh "Trampolining"] OR [mh "Exercise Therapy"] OR trampoline*:ti,ab,kw OR "trampoline training":ti,ab,kw OR "trampoline exercise*":ti,ab,kw OR "mini-trampoline":ti,ab,kw OR "rebound exercise":ti,ab,kw OR "rebound training":ti,ab,kw OR "rebound therapy":ti,ab,kw AND [mh "Motor Skills"] OR [mh "Motor Activity" OR [mh "Physical Fitness"] OR [mh "Muscle Strength"] OR [mh "Postural Balance"] OR [mh Gait] OR "motor ability":ti,ab,kw OR "motor performance":ti,ab,kw OR "motor function":ti,ab,kw OR "physical performance":ti,ab,kw OR "functional performance":ti,ab,kw OR "exercise capacity":ti,ab,kw OR "muscle strength":ti,ab,kw OR "muscle power":ti,ab,kw OR balance:ti,ab,kw OR "postural control":ti,ab,kw OR gait:ti,ab,kw AND [mh Humans] OR adult*:ti,ab,kw OR child*:ti,ab,kw OR adolescen*:ti,ab,kw OR athlete*:ti,ab,kw OR patient*:ti,ab,kw AND [mh "Randomized Controlled Trials"] OR [mh "Clinical Trials as Topic"] OR RCT:ti,ab,k OR random*:ti,ab,kw OR trial*:ti,ab,kw OR "controlled study":ti,ab,kw OR "control group":ti,ab,kw OR intervent*:ti,ab,kw |

**Quality Assessment**

| **authors** | **D1** | **D2** | **D3** | **D4** | **D5** | **Overall** |
| --- | --- | --- | --- | --- | --- | --- |
| Azab et al 2022 | Some concerns | High | High | Low | Low | High |
| Lourenço et al 2015 | High | High | Low | Some concerns | Some concerns | High |
| Aalizadeh et al 2016 | Some concerns | High | Low | Some concerns | Low | Some concerns |
| Giagazoglou et al 2013 | Low | High | Low | Low | Low | Some concerns |
| AL-Nemr & Kora 2024 | Some concerns | High | Some concerns | Some concerns | Low | High |
| Belaiba et al 2024 | Some concerns | High | Low | Low | Low | High |
| Haghigh et al 2019 | Some concerns | Some concerns | Low | Low | Low | Some concerns |
| Kiran Pt et al 2022 | High | High | Low | Some concerns | Low | High |
| Touali et al 2025 | High | High | Low | Low | Low | High |
| Sanglakh et al 2017 | Some concerns | High | Low | Low | Low | High |
| Yamanishi et al 2025 | Low | Some concerns | Low | Low | Low | Some concerns |
| Abd-Elmonem et al 2018 | Some concerns | High | Low | Low | Low | High |
| Sulaiman et al 2022 | Some concerns | High | Some concerns | Some concerns | Low | High |
| Khan et al 2025 | Some concerns | High | Low | Some concerns | Low | High |
| Tekin et al 2025 | Low | High | Low | Low | Low | High |

**Static balance**

Egger’s Test Funnel Plot for Publication Bias Assessment.


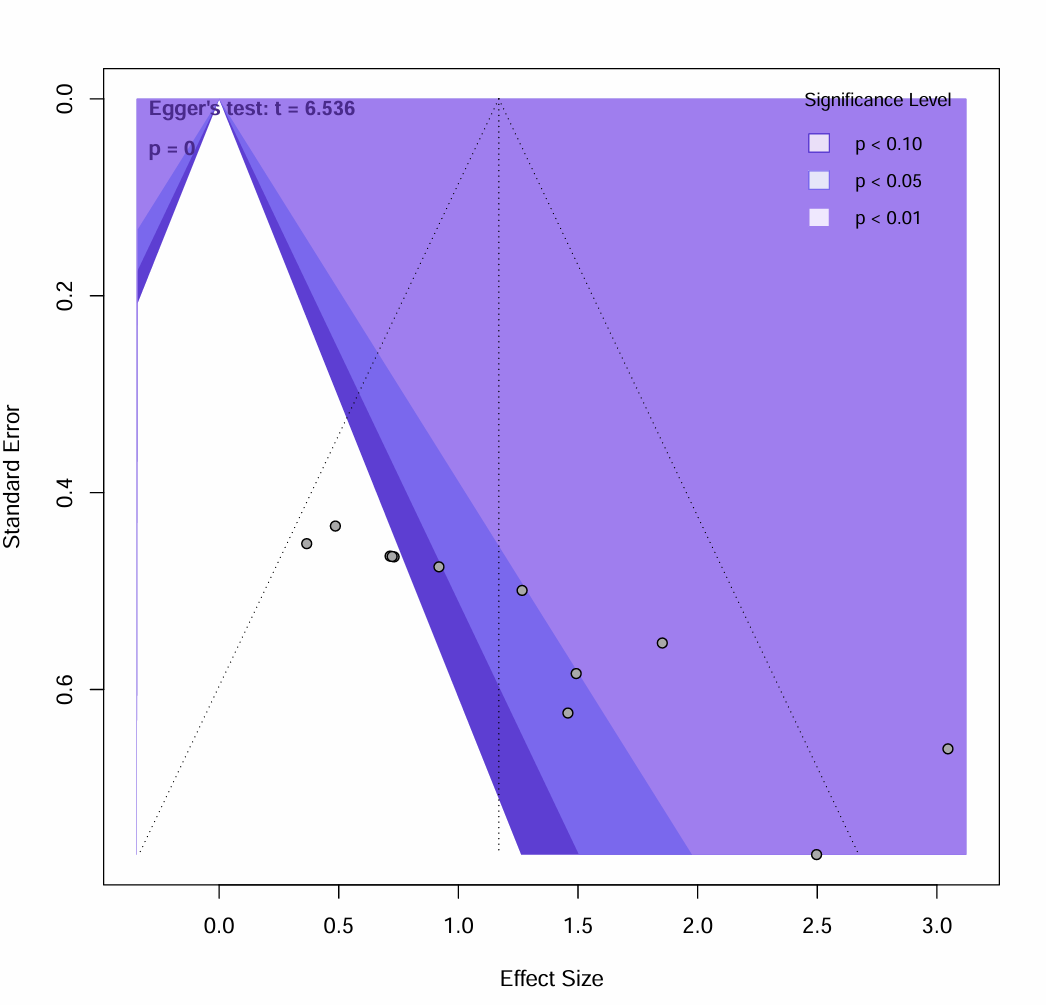


Influence diagnostics for included studies using standardized residuals and Cook’s distance.


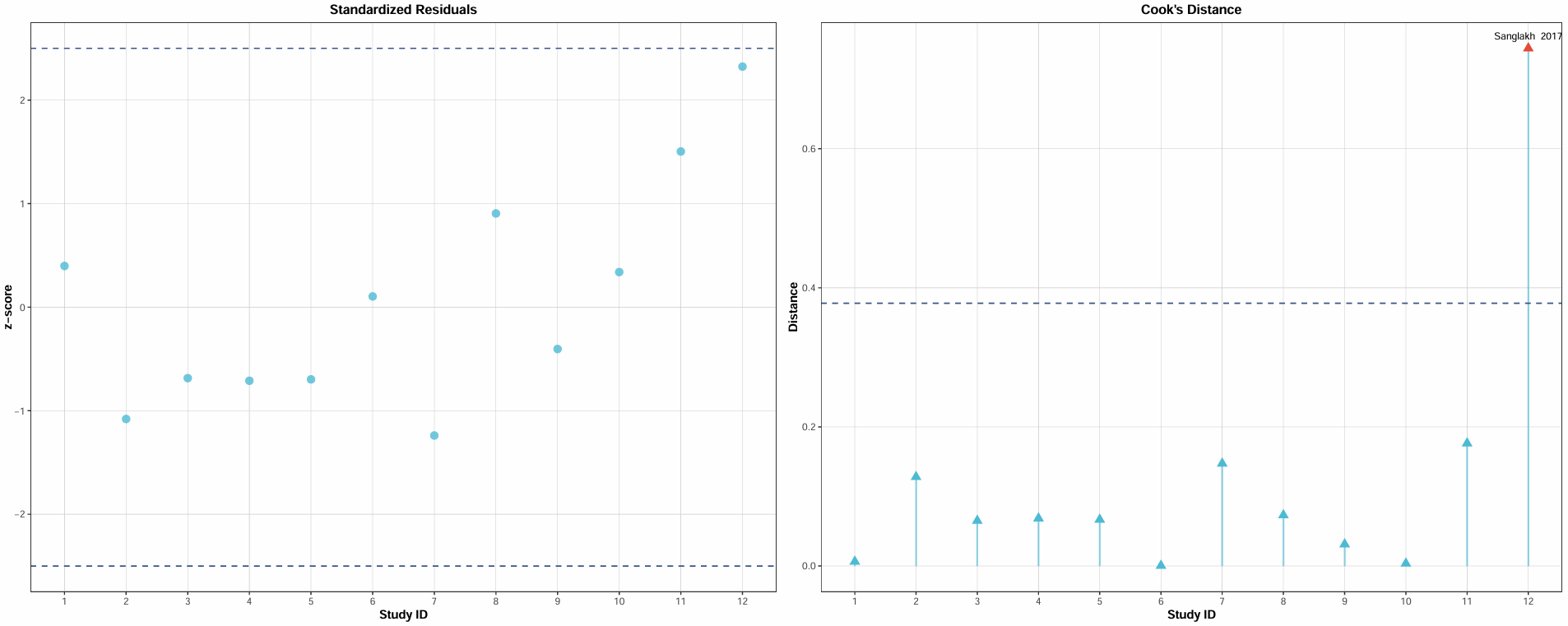


Figure of the Initial Sensitivity Analysis.


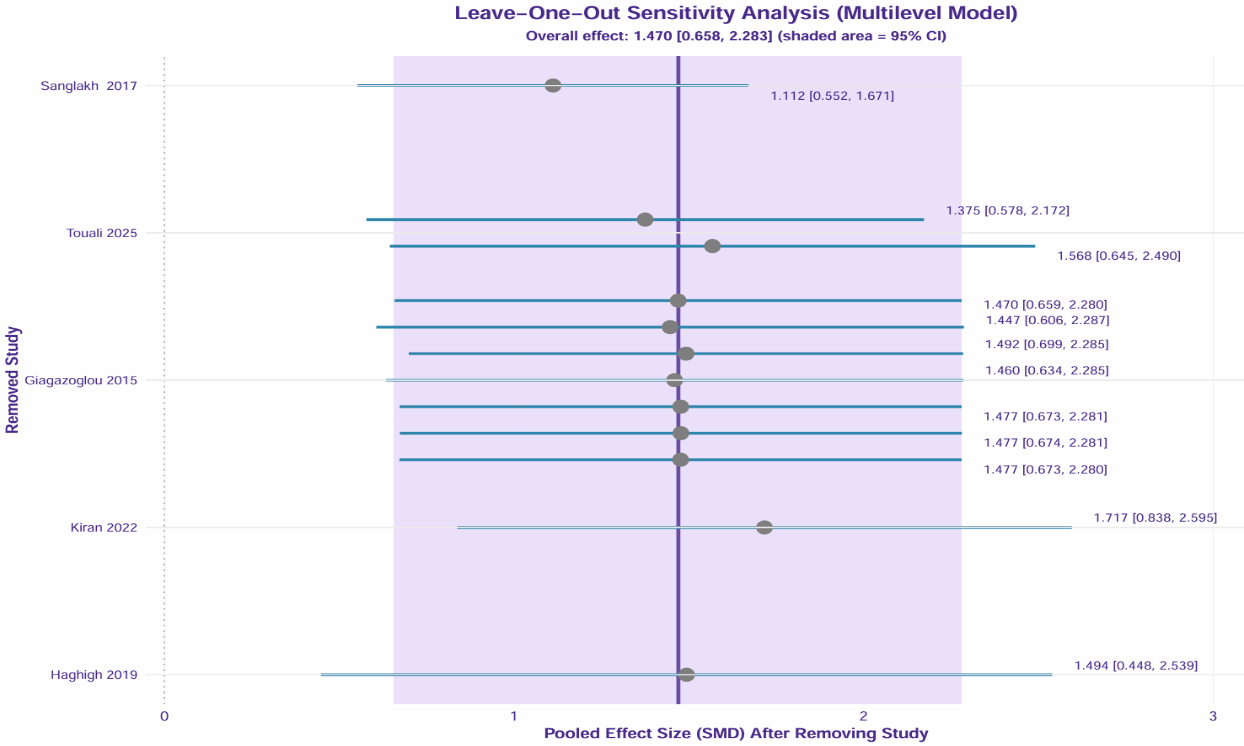


Trim-and-Fill Funnel Plot


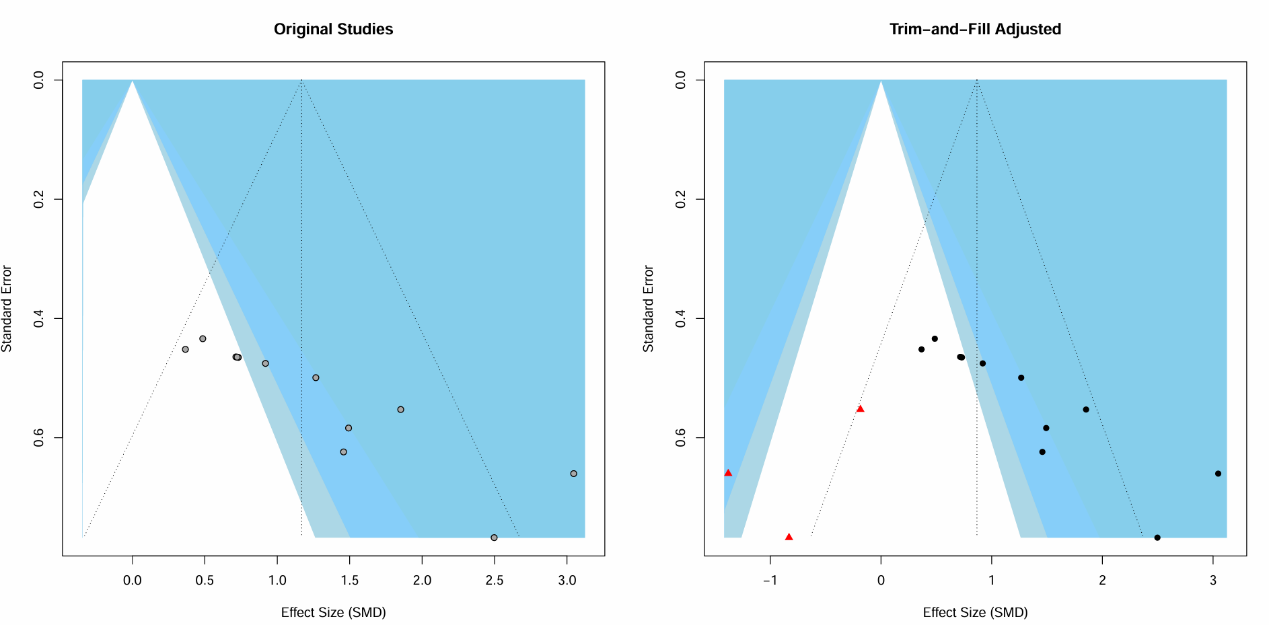


Summary of Findings — The effect of trampoline training on static balance ability in children and adolescents with special needs.

| GRADE Domain | Judgment | Rationale (Strictly Based on the Original Article) |
| --- | --- | --- |
| Risk of Bias | Serious (Downgraded by 1 level) | Among the 5 included studies for static balance, most had some concerns in the randomization process (D1) and high risk in deviations from intended interventions (D2), with 4 studies rated as high risk in D2. Missing outcome data (D3) and selective reporting (D5) were mostly rated as low risk, while outcome measurement (D4) showed some concerns in a minority of studies. Overall, the risk of bias for static balance was judged as some concerns to high. |
| Inconsistency | Serious (Downgraded by 1 level) | Heterogeneity was extremely high（I² = 79.1%，P = 0.0075），Although subgroup analysis identified the optimal intervention scheme, heterogeneity was not fully explained. |
| Indirectness | Not downgraded | The population (children and adolescents with special needs), intervention (trampoline training), comparators, and static balance outcomes were directly aligned with the research question, with no concerns regarding applicability. |
| Imprecision | Not downgraded | A total of 5 studies (228 participants) were included, with a pooled effect size of SMD = 1.47 (95%CI: 0.66~2.28, P <0.001). The confidence interval did not include 0, and the sensitivity analysis showed stable results. |
| Publication Bias | Not downgraded | Egger’s test was significant (t = 6.54, p < 0.001), but trim-and-fill correction remained significant (adjusted SMD = 0.87, 95% CI 0.34–1.39, P = 0.0012), suggesting the direction/evidence for static balance is comparatively more robust after bias adjustment. |

**Dynamic balance**

Egger’s Test Funnel Plot for Publication Bias Assessment.


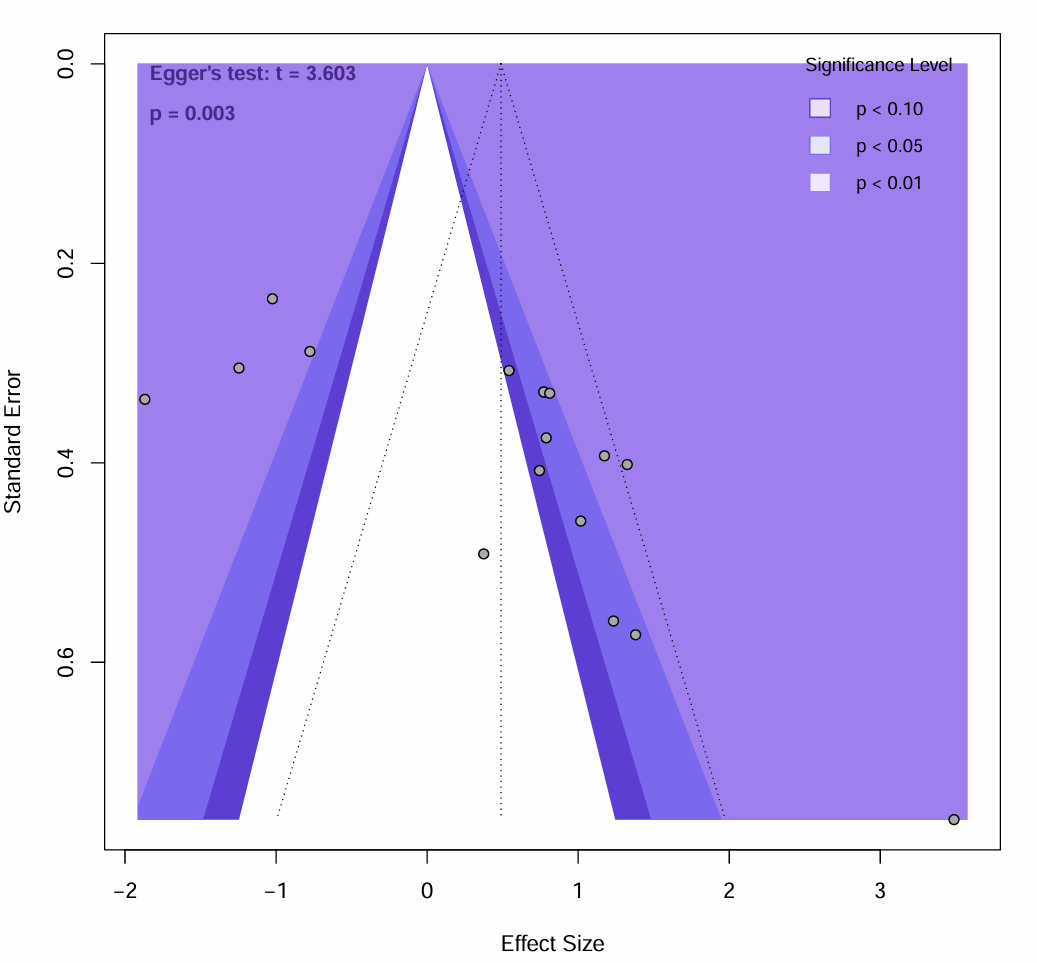


Influence diagnostics for included studies using standardized residuals and Cook’s distance.


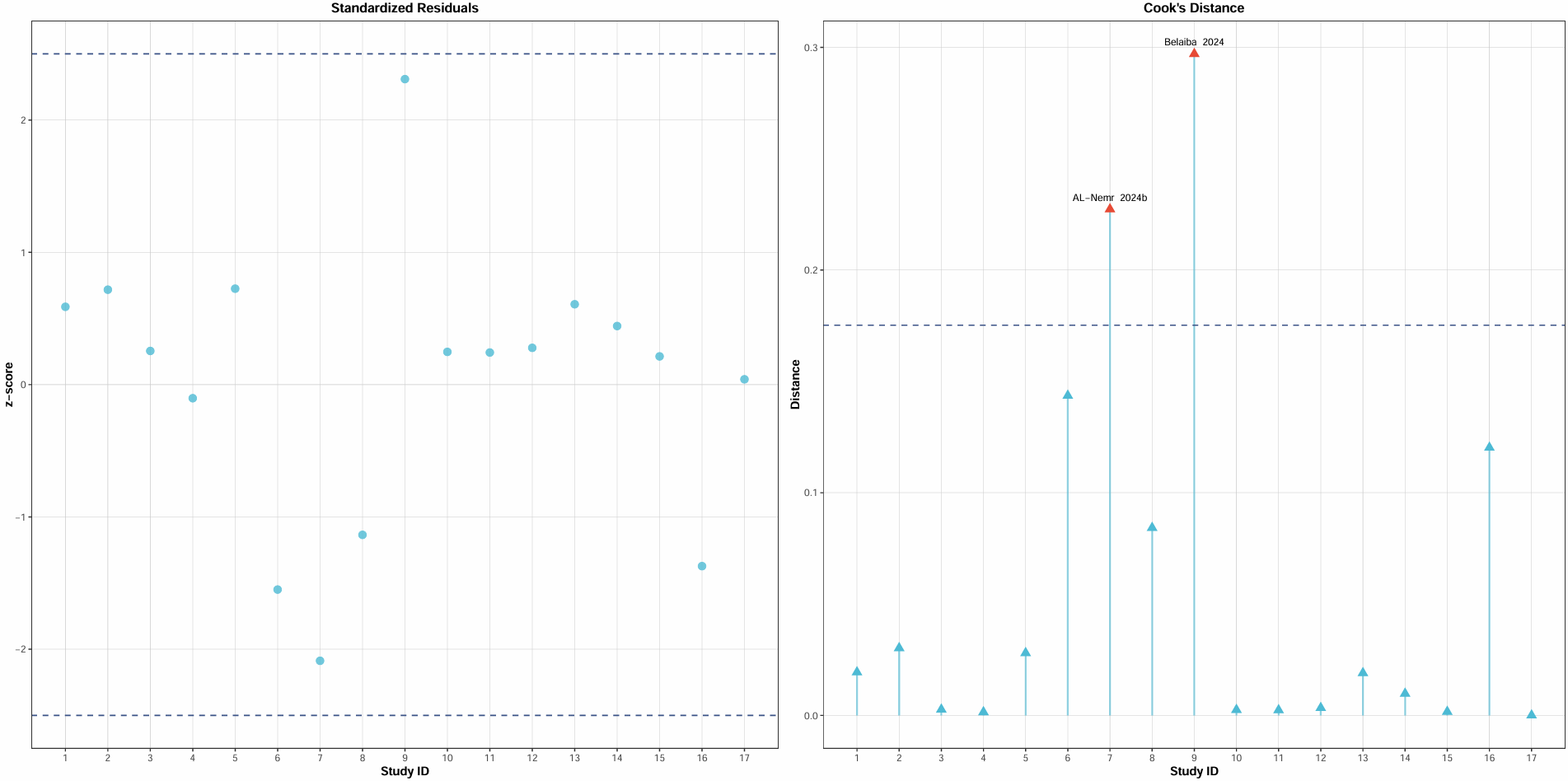


Figure of the Initial Sensitivity Analysis.


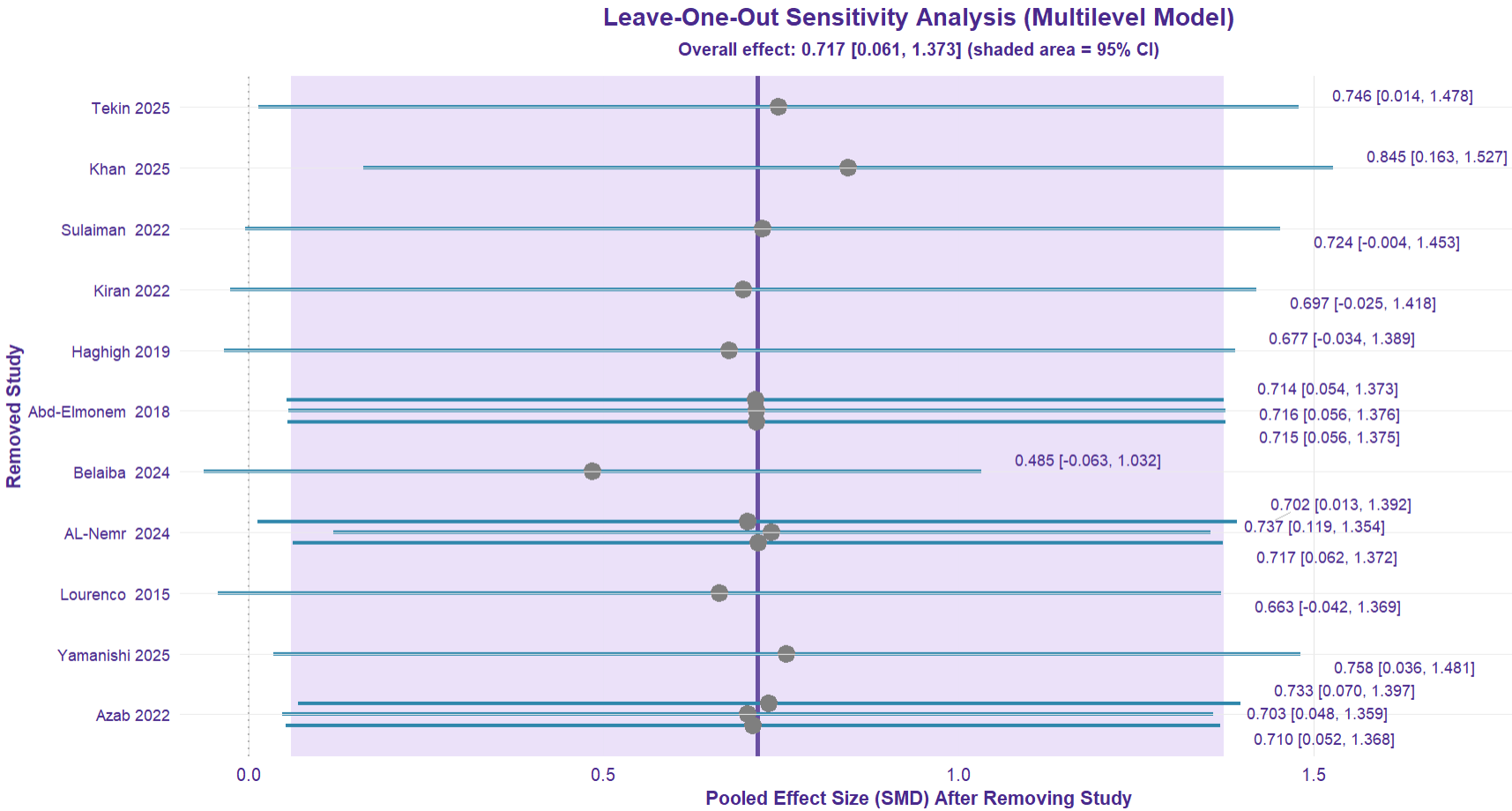


Trim-and-Fill Funnel Plot


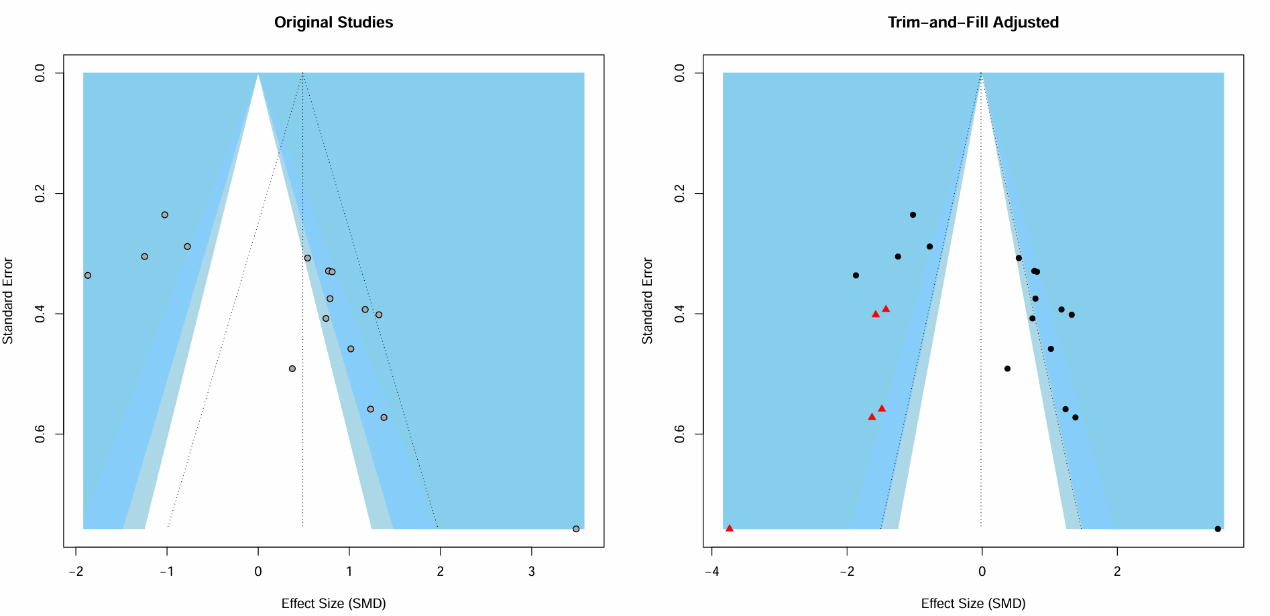


Summary of Findings — Effects of trampoline training on dynamic balance ability of children and adolescents with special needs

| GRADE Domain | Judgment | Rationale (Strictly Based on the Original Article) |
| --- | --- | --- |
| Risk of Bias | Serious (Downgraded by 1 level) | Among the 11 included studies for dynamic balance, most had some concerns in the randomization process (D1) and were rated at high risk in deviations from intended interventions (D2), with the majority of studies judged as high risk in D2. Missing outcome data (D3) and selective reporting (D5) were mostly rated as low risk, while outcome measurement (D4) showed some concerns in several studies. Overall, the risk of bias for dynamic balance was judged as high, largely driven by the high-risk D2 domain. |
| Inconsistency | Serious (Downgraded by 1 level) | Heterogeneity was extremely high(I² = 90.5%, P < 0.001). Although subgroup analysis identified the optimal intervention scheme, heterogeneity was not fully explained. |
| Indirectness | Not downgraded | Population/intervention/comparator/outcome are directly relevant to the review question. |
| Imprecision | Not downgraded | A total of 11 studies (612 participants) were included, with a pooled effect size of SMD = 0.72 (95%CI: 0.06~1.37, P = 0.032). The confidence interval did not include 0, and the sensitivity analysis showed stable results. |
| Publication Bias | Not downgraded | Egger’s test was significant (t = 3.60, p = 0.003), and trim-and-fill correction attenuated the effect to near zero and non-significant (adjusted SMD = −0.02, 95% CI −0.64–0.61, P = 0.9556), indicating strong sensitivity to publication bias/small-study effects. |

Dynamic equilibrium sequential test analysis and cumulative meta-analysis diagram.


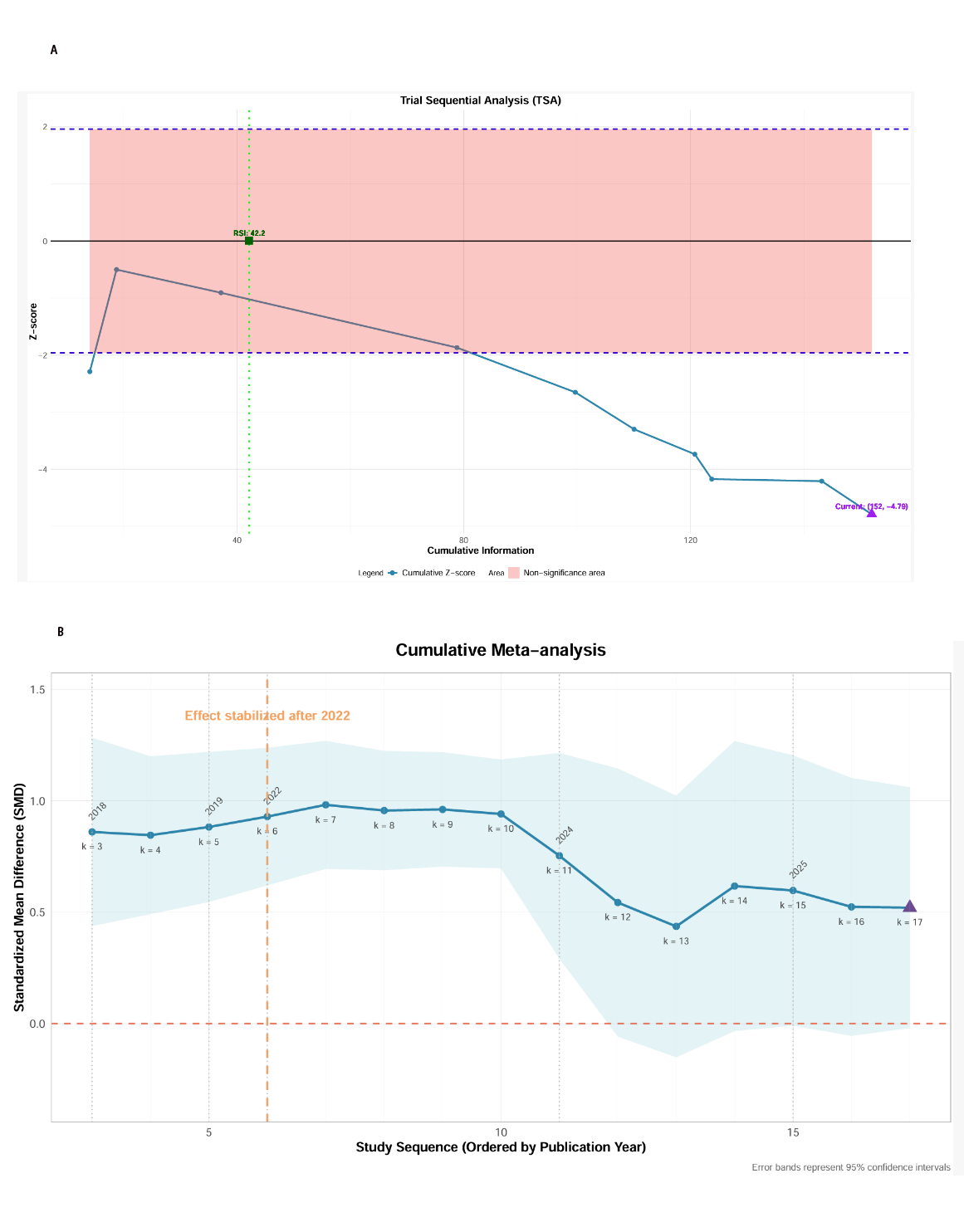


**Lower limb strength**

Egger’s Test Funnel Plot for Publication Bias Assessment.


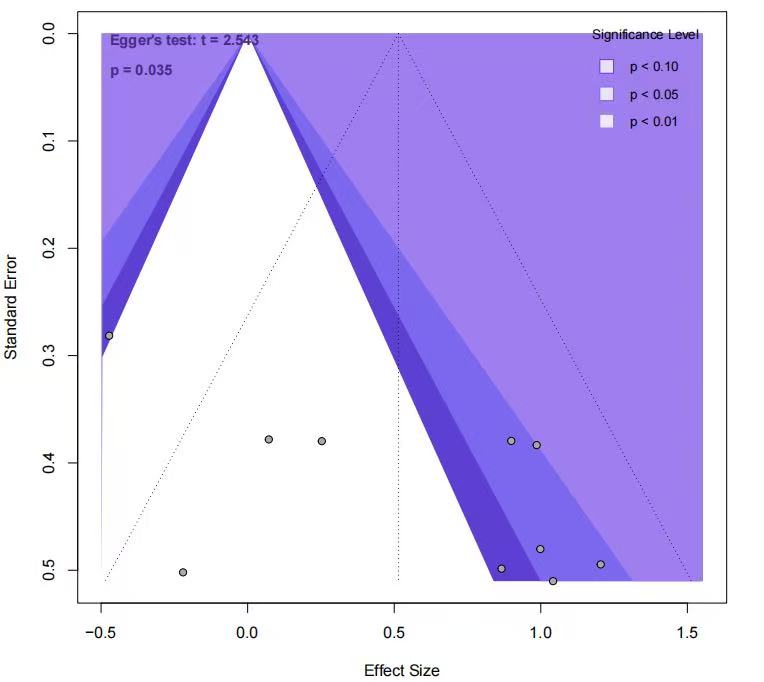


Influence diagnostics for included studies using standardized residuals and Cook’s distance.


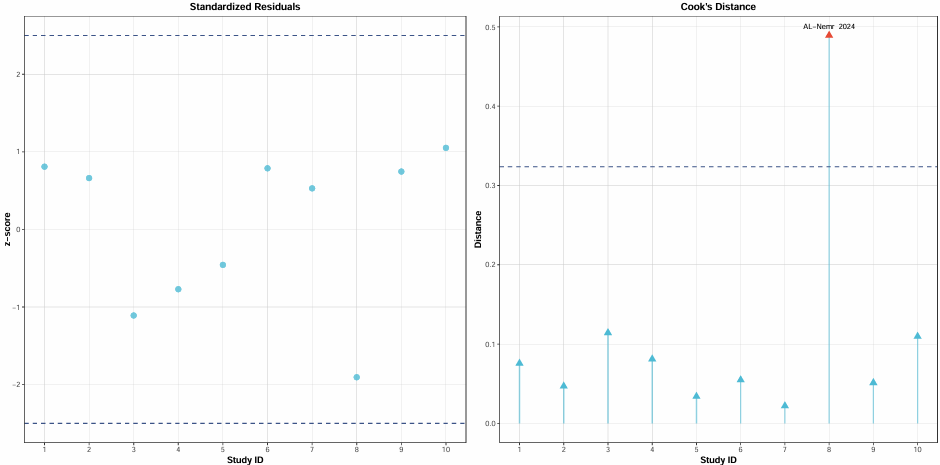


Figure of the Initial Sensitivity Analysis.


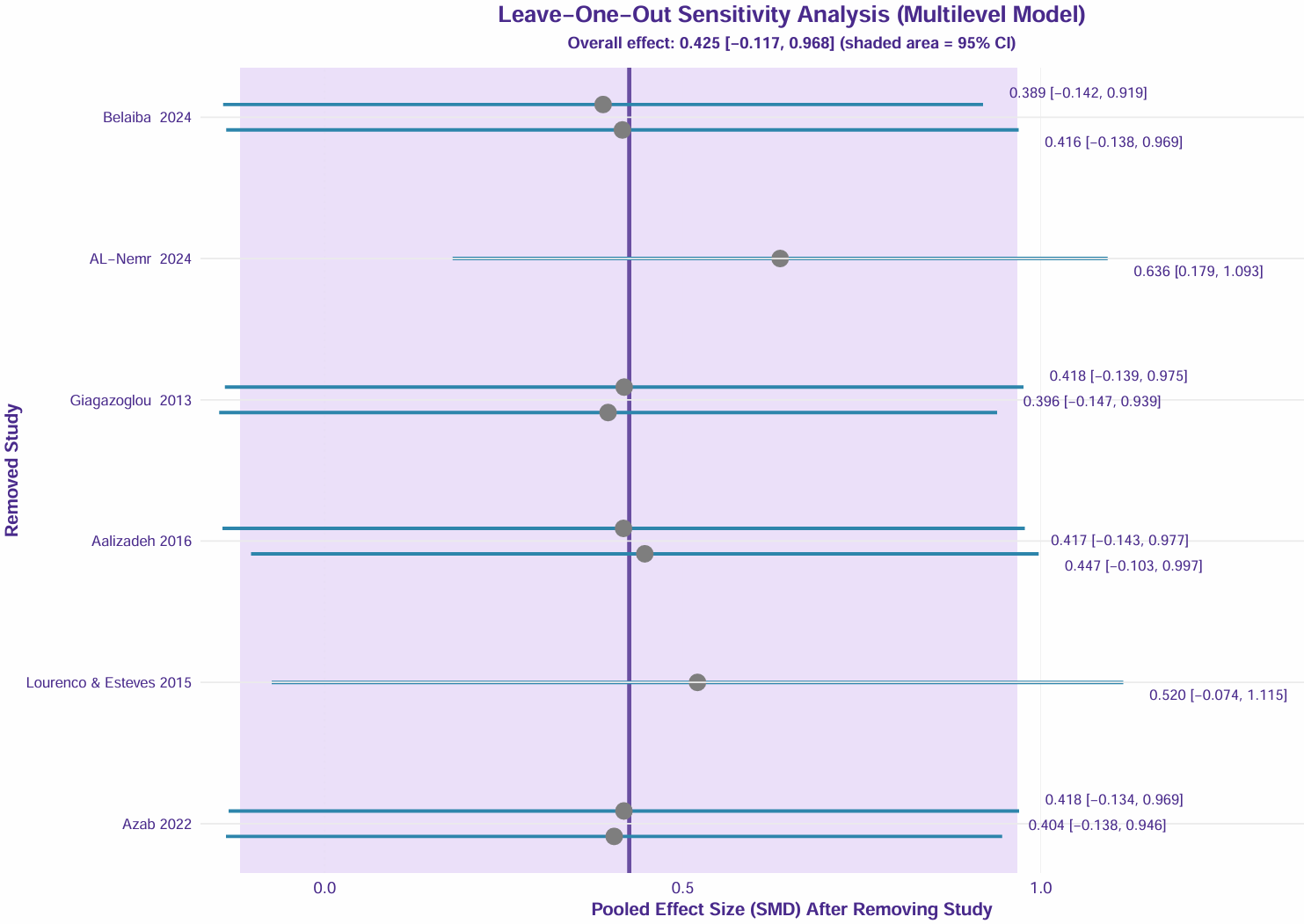


Trim-and-Fill Funnel Plot


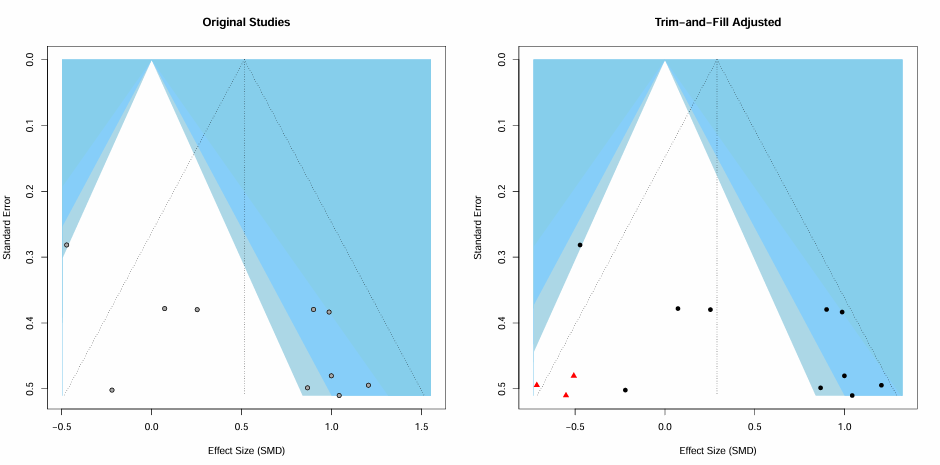


## Summary of Findings — Effects of trampoline training on lower limb strength ability in children and adolescents with special needs

| GRADE Domain | Judgment | Rationale (Strictly Based on the Original Article) |
| --- | --- | --- |
| Risk of Bias | Serious (Downgraded by 1 level) | Among the 6 included studies, most had *some concerns* in the randomization process (D1) and *high risk* in deviations from intended interventions (D2), with all 6 studies rated as high risk in D2. Missing outcome data (D3) and selective reporting (D5) were mostly rated as *low risk*, whereas outcome measurement (D4) showed *some concerns*. Overall, the risk of bias for lower-limb strength was judged as *high*. |
| Inconsistency | Serious (Downgraded by 1 level) | Substantial heterogeneity for lower-limb explosive power (I² = 82%, P = 0.006). |
| Indirectness | Not downgraded | The evidence directly addresses the target population and the intended outcome (lower-limb strength/explosive power) under trampoline-based interventions. |
| Imprecision | Serious (Downgraded by 1 level) | A total of 6 studies, with a pooled effect size of SMD = 0.43 (95% CI −0.12 to 0.97, p = 0.125). The confidence interval crossed 0, and the sensitivity analysis results were unstable. |
| Publication Bias | Serious (Downgraded by 1 level) | Egger’s test suggested publication bias (t = 2.54, p = 0.035), and trim-and-fill further attenuated the estimate to non-significance (adjusted SMD = 0.29, 95% CI −0.10–0.68, P = 0.1428). |
